# A cohort study of neurodevelopmental disorders and/or congenital anomalies using high resolution chromosomal microarrays in southern Brazil highlighting the significance of ASD

**DOI:** 10.1038/s41598-024-54385-2

**Published:** 2024-02-14

**Authors:** Tiago Fernando Chaves, Maristela Ocampos, Ingrid Tremel Barbato, Louise Lapagesse de Camargo Pinto, Gisele Rozone de Luca, Jorge Humberto Barbato Filho, Priscila Bernardi, Yara Costa Netto Muniz, Angelica Francesca Maris

**Affiliations:** 1 Laboratório de Polimorfismos Genéticos (LAPOGE), Universidade Federal de Santa Catarina, Florianópolis, SC Brazil; 2 Universidade Federal de Santa Catarina, Florianópolis, SC Brazil; 3 Laboratory Neurogene (former), Florianopolis, SC Brazil; 4 Present Address: Mercolab Diagnóstica (actual), Florianopolis, SC Brazil; 5 Laboratory Neurogene, Florianópolis, SC Brazil; 6 Children’s Hospital Joana de Gusmão, Florianópolis, SC Brazil; 7 University Hospital Professor Polydoro Ernani de São Thiago, Florianópolis, SC Brazil

**Keywords:** Autism, Congenital anomalies, LCSH, Copy number variations, Neurodevelopmental disorders, Chromosomal microarrays, Brazil, Clinical genetics, Consanguinity, Cytogenetics, Development, Genetic hybridization, Genome, Genomic instability, Medical genetics, Population genetics

## Abstract

Chromosomal microarray (CMA) is the reference in evaluation of copy number variations (CNVs) in individuals with neurodevelopmental disorders (NDDs), such as intellectual disability (ID) and/or autism spectrum disorder (ASD), which affect around 3–4% of the world’s population. Modern platforms for CMA, also include probes for single nucleotide polymorphisms (SNPs) that detect homozygous regions in the genome, such as long contiguous stretches of homozygosity (LCSH). These regions result from complete or segmental chromosomal homozygosis and may be indicative of uniparental disomy (UPD), inbreeding, population characteristics, as well as replicative DNA repair events. In this retrospective study, we analyzed CMA reading files requested by geneticists and neurologists for diagnostic purposes along with available clinical data. Our objectives were interpreting CNVs and assess the frequencies and implications of LCSH detected by Affymetrix CytoScan HD (41%) or 750K (59%) platforms in 1012 patients from the south of Brazil. The patients were mainly children with NDDs and/or congenital anomalies (CAs). A total of 206 CNVs, comprising 132 deletions and 74 duplications, interpreted as pathogenic, were found in 17% of the patients in the cohort and across all chromosomes. Additionally, 12% presented rare variants of uncertain clinical significance, including LPCNVs, as the only clinically relevant CNV. Within the realm of NDDs, ASD carries a particular importance, owing to its escalating prevalence and its growing repercussions for individuals, families, and communities. ASD was one clinical phenotype, if not the main reason for referral to testing, for about one-third of the cohort, and these patients were further analyzed as a sub-cohort. Considering only the patients with ASD, the diagnostic rate was 10%, within the range reported in the literature (8–21%). It was higher (16%) when associated with dysmorphic features and lower (7%) for "isolated" ASD (without ID and without dysmorphic features). In 953 CMAs of the whole cohort, LCSH (≥ 3 Mbp) were analyzed not only for their potential pathogenic significance but were also explored to identify common LCSH in the South Brazilians population. CMA revealed at least one LCSH in 91% of the patients. For about 11.5% of patients, the LCSH suggested consanguinity from the first to the fifth degree, with a greater probability of clinical impact, and in 2.8%, they revealed a putative UPD. LCSH found at a frequency of 5% or more were considered common LCSH in the general population, allowing us to delineate 10 regions as potentially representing ancestral haplotypes of neglectable clinical significance. The main referrals for CMA were developmental delay (56%), ID (33%), ASD (33%) and syndromic features (56%). Some phenotypes in this population may be predictive of a higher probability of indicating a carrier of a pathogenic CNV. Here, we present the largest report of CMA data in a cohort with NDDs and/or CAs from the South of Brazil. We characterize the rare CNVs found along with the main phenotypes presented by each patient and show the importance and usefulness of LCSH interpretation in CMA results that incorporate SNPs, as well as we illustrate the value of CMA to investigate CNV in ASD.

## Introduction

Neurodevelopmental disorders (NDDs) predominantly encompass developmental delay (DD), intellectual disability (ID), and/or autism spectrum disorders (ASD), impacting approximately 3–4% of the global population^1,2^. These conditions are classified as non-syndromic when they occur in isolation and syndromic when they co-occur with dysmorphisms or evident congenital anomalies (CAs)^3^.

With strong genetic underpinnings, ASD holds great significance within the realm of NDDs due to its high prevalence and increasing impact on individuals, families, and communities. The disorder's heterogeneity spans a wide spectrum of symptoms and severity, usually accompanied by co-occurring conditions, being characterized by impairment in social interaction and communication. According to the Diagnostic and Statistical Manual of Mental Disorders—Fifth Edition (DSM-5), we can understand the deficits in social interactions and social communications of individuals with ASD based on three aspects: socio-emotional reciprocity; non-verbal communicative behaviors used for social interaction, development, maintenance and understanding of relationships; and restricted behaviors, such as repetitive patterns exhibited as movements, repetitive use of objects or speech, unalterable routines or ritualized behaviors (verbal or non-verbal), fixation on singular interests, and abnormal response to variations in sensory aspects of the environment^4^. Based on common deficits, the DSM-5 defines the current diagnosis of ASD that now, along with those of autistic disorder (classical autism), also incorporates the diagnoses of childhood disintegrative disorder, pervasive developmental disorder without other specification, and Asperger's syndrome.

Sometimes ASD is the main diagnosis, sometimes it is comorbid to other NDDs such as ID, frequent in the autistic spectrum. It can also be present in syndromic conditions when apparent dysmorphic features (DF) for their potential CAs are present^3^.

It is estimated that ASD presents a heritability between 0.5 and 0.9%^5,6^. A recent review covering 74 studies with 30,212,757 participants concluded an estimated global prevalence of ASD of 0.6%. It is highest in America (1%), Africa (1%) and Australia (1.7%)^7^. The prevalence of ASD worldwide has increased in recent decades, for example in the USA, the Centers for Disease Control and Prevention reported that the overall prevalence of ASD was 1,5% in 2010, 1.4% in 2012, 1.7 in 2014 and 1.9 in 2016, 2.3 in 2018 (CDC). The overall prevalence of ASD in Europe and Asia has also been gradually increasing^8,9^. In Brazil, as well as in Latin America in general, epidemiological data on the prevalence of ASD are scarce. A single study carried out in the Southeast region of Brazil in 2011, found an estimated prevalence of 0,3%^10^, however, it is believed to be an underestimation due to methodological issues. If we apply the prevalence of 1% estimated for the American population to the Brazilian population (214 million), ASD should affect approximately 2 million individuals^11^.

Genetic and/or genomic factors such as single nucleotide polymorphisms (SNPs) and CNVs^12–17^ have been suggested as the etiological cause in 50–60% of cases of ASD^18^. The SFARI Gene^6^, one of the leading and constantly updated genetic databases on ASD, associates 1,262 genes and 2,290 CNVs, including those with rare frequency, to the condition (data from December 2022).

CNVs are structural variations in the DNA that involve gains or losses of large segments of genetic material (from hundreds to several million base pairs) that may be inherited or occur spontaneously during the formation of egg or sperm cells and can affect gene dosage, causing loss of function, haploinsufficiency, or overexpression of genes^19^. Specific CNVs have been shown to cause or increase the likelihood of developing certain NDDs such as ID, ASD, schizophrenia, as well as CAs. However, most people with CNVs do not have developmental disorders and for many CNVs related to disorders the presence of the CNV per se does not implicate necessarily the presence of the disorder, because their penetrance and expression is impacted by other genetic and/or by environmental factors, which makes their interpretation challenging.

For over a decade, Chromosomal microarray (CMA) technologies have been clinically recommended as the primary cytogenetic diagnostic test for investigating patients with NDDs^20^ and in 2020 the ACMG reinforced this statement, along with a more detailed guidance on interpreting results^21^.

Most modern microarray platforms along with genome-wide oligonucleotide probes (depending on the CMA design) also integrate high-density SNP probes, that test for single nucleotide changes in DNA sequences, allowing to detect regions of homozygosity that can be associated with disease or other traits like ancestry.

Long contiguous stretches of homozygosity (LCSHs) are relatively common in the general population and can occur due to the chance of unions among individuals with a common ancestor, in these cases they rarely are related to disease, likely characterizing regions of low recombination in the genome^22,23^. However, larger LCSHs can also reveal consanguinity among parents, uniparental disomy (UPD) or homologous recombinational DNA repair events and therefore be associated with an increased risk for certain genetic disorders, particularly those caused by recessive genetic mutations. In population studies, the minimal thresholds for calling LCSH are usually set around 0.5–1.0 Mbp, while in clinical analysis, minimal thresholds are more conservatively set at 3–10 Mbp^24^.

The presence of multiple large LCSH ≥ 5 Mbp, distributed throughout several chromosomes suggests consanguinity between the individual’s biological parents, increasing the chance of inheritance of recessive monogenic disorders. However, when large LCSH(s), reside in only one chromosome, this can reflect correction of meiotic or early post meiotic errors that resulted in total or partial uniparental disomy (UPD). UPD occurs when a person receives the two copies of a chromosome, or part of a chromosome, from only one parent^25^. The two copies can be of maternal (UPDmat) or paternal (UPDpat) origin. An UPD is not necessarily pathogenic, however it is an important cause of genetic disease because several genes suffer genomic imprinting, which silences one allele of the chromosomal pair in a gender-specific manner and a series of imprinting disorders cause NDs associated with ID, autistic behavior, DD and seizures. Examples include the Angelman’s syndrome (UPD (15) pat), Prader-Willi syndrome (UPD (15) mat), Beckwith-Wiedemann syndrome (UPD (11) pat), Silver-Russell syndrome (UPD (7) mat), Temple syndrome (UPD (14) mat) and Kagami-Ogata syndrome (UPD (14) pat)^26^. Even when not affecting imprinted genes, the UPD can uncover recessive mutations in the uniparental homozygotic regions, for which the sole transmitting parent of this region was heterozygous.

Whole chromosome UPDs can arise as consequence of the correction of a meiotic segregation error that resulted in a monosomic or a trisomic zygote, by duplicating the only chromosome present in the monosomic zygote or by losing one of the exceeding chromosomes in case of trisomy. In the monosomy rescue both chromosomes of the pair will be from only one progenitor and completely homozygous (isodisomic) whether in the trisomy rescue the UPD only occurs when the two chromosomes that were retained are from the same progenitor. In later case they can be totally isodisomic when the meiotic non-disjunction of the two sister chromatids occurred in meiosis II, however, when the meiotic error occurred in meiosis I, because of the homologous chromosomal recombination they will be partially iso/heterodisomic (one or more LSCHs on the chromosome) or completely heterodisomic (not originating homozygous regions) since the outer sister chromatids do not recombine^27,28^. Segmental UPDs can have complex causes, like rescue of a partial trisomy caused by translocated chromosomes, DNA double-strand breaks or others involving a replicative DNA repair mechanism^28–32^.

The aims of this study included establishing the overall diagnostic rate of CMA in our settings, to verify the contribution of LCSH, the significance of patients with ASD phenotypes, to see if there is a difference in the diagnostic yield when considering only those with ASD phenotypes, and to provide detailed genetic data of known causal CNVs and/or of other rare, possibly causal, CNVs identified in the cohort.

## Methods

### Ethical aspects

The research project was reviewed and approved by the Research Ethics Committee of the Hospital Infantil Joana de Gusmão, the children's hospital in Florianópolis-SC, Brazil, under the reference number 2339104. We further declare that the study was conducted accordance with ethical standards and guidelines, set forth in resolution No. 466/12 of the Brazilian National Health Council. Patients or their caregivers provided informed consent to participate in the study. In cases where it was not possible to contact the patient for justifiable reasons (such as loss of contact information), the data was still used, and a Justification of Absence of Consent was signed by the research team. The team committed to maintaining the confidentiality and privacy of the patients whose data and/or information was collected in the records.

### Cohort

The aim of this study was to investigate a significant cohort with developmental disorders from South Brazil. We collected a total of 1120 chromosomal microarray (CMA) read files that were performed by the Laboratório Neurogene in Florianópolis, Santa Catarina, Brazil, upon request by medical geneticists and neurologists for investigative/diagnostic purposes, primarily from the Joana de Gusmão Children's Hospital, but also from MDs from the University Hospital Professor Polydoro Ernani de São Thiago and from private clinics in Florianópolis, State of Santa Catarina, between 2013 and 2019. These include also 420 previously published cases^28,33^. Furthermore, 68 out of 1120 cases were excluded because they belonged to unaffected family members and 40 cases were excluded from the statistics of developmental disorders due to insufficient clinical information. The analyzed sample, therefore, consists of CMA read files and available clinical data from 1,012 patients, primarily children with neurodevelopmental disorders, from southern Brazil.

For analysis of the significance of ASD in our cohort, we established a sub-cohort where we included every patient of the cohort where the clinical phenotype specifically mentioned ASD, autistic disorder (classical autism), childhood disintegrative disorder, pervasive developmental disorder without other specification or Asperger's syndrome as the main reason for referral to testing or as one of the phenotypes of a broader spectrum. We call "syndromic autism" those patients that had dysmorphic features/congenital anomalies (accompanied or not by intellectual disability) mentioned within their clinical phenotypes. In non-syndromic cases we have autism with intellectual disability and what we call "isolated autism", which would be the non-syndromic autism without intellectual disability. The ASD sub-cohort refers to 333 patients from the south of Brazil, of which 134 are part of a previously published study^33^, for which CMA reading files and clinical data were available.

### Collection of clinical data

To establish a correlation between the phenotype and potential causal genes, we gathered the required phenotypic/clinical data in the exam request form and, when possible, supplemented with direct information by their medical doctors. This was done through a questionnaire that asked information about the individual's clinical presentation, behavior, history of physical exams, previous genetic and metabolic tests results, and prescription medication. No new appointments were arranged with the patients for this study, and clinicians retrieved most of the data from their medical records.

### Genomic analysis

The investigative CMA platforms used were CytoScan 750K (59%) and CytoScan HD (41%) and the resulting files were analysed using the Chromosome Analysis Suite (ChAS) Affymetrix software^4^, which is based on the reference genome sequence of the University of California, Santa Cruz database (https-//genome.ucsc.edu/cgi-bin/hgGateway) using the human genome version of February 2009 (GRCh37/hg19). The analysis was retrospective, with the use of the CMA runs obtained from a clinical diagnostic laboratory, with previous consent of the patients.

Typically, the filter criteria for interpreting CNVs for diagnostic purposes are sizes larger than 100 Kbp for deletions and larger than 150 Kbp for duplications, both containing at least 50 markers, according to ACMG recommendations^19,20^. However, since this is a research study, that aims to identify potential new genes involved in developmental disturbances, we reduced the filter parameters to > 10 Kbp for deletions and for duplications, both with at least ten markers. To interpret the CNVs, we followed the latest recommendations of the ACMG and the Clinical Genome Resource^21^.

### CNVs interpretation and classification

To interpret CNVs, regarding their function, dosage effects (known haploinsufficiency or overexpression studies) and effects of mutations, the UCSC Genome Browser with integrated databases was widely used, mainly ClinVar (NCBI), DECIPHER (Database of Chromosomal Imbalance and Phenotype in Humans using Ensembles Resources), DGV (Database of Genomic Variants), OMIM (Online Mendelian Inheritance in Man), ISCA (International Standard Cytogenomic Array), dbGaP (Database of Genotypes and Phenotype), dbVAR (Database of Large Scale Genomic Variants), ECARUCA (European Cytogeneticists Association Register of Unbalanced Chromosome Aberrations), PUBMED (Public Medline), ClinGen (Clinical Genome Resource), MGI (Mouse Genome Informatics Database, from The Jackson Laboratory), SFARI (Simons Foundation Autism Research Initiative) and the private database CAGdb (Cytogenomics Array Group CNV Database). We also used the the Franklin platform^34^, based on Artificial Intelligence, as a tool for classification and interpretation of genomic variants using scores^21^.

The variants were classified into four types according to clinical interpretation as benign variants, variants of uncertain significance (VUS), likely pathogenic VUS (LPCNVs), or pathogenic variants (PCNVs), and the result in each case was assigned based on the CNVs of greatest clinical relevance detected in the genome of the patients^21^.

Variables like location, type and size of each CNV, the CNV classification, number of CNVs detected for each individual, age, gender, clinical descriptions (phenotypes), previous genetic testing results (karyotype, fragile X, etc.), and other relevant known clinical data to which we had access, were compiled (with coded identification) into simple Excel sheet for data handling with the R software [version 3.4.2] (R Foundation for Statistical Computing). This was done to understand the phenotypic frequency, the diagnostic rates, the average age and the gender distribution in the cohort, the frequency of genomic changes in each chromosome and to find if there are any phenotypic clues related to a higher diagnostic probability by CMA (predictive phenotypes of a higher chance to be related to a pathogenic CNV), that eventually could allow selecting the cases that would benefit the most using CMA as a first-line test in settings of financial shortage.

### Statistics

In the study, in addition to the descriptive biostatistical analysis, the univariate analysis (Fisher's test) was applied to identify eventual predictive phenotypes for a higher diagnostic result (greater chance of having a pathogenic CNV). To compare the mean sizes, amounts of covered genes and quantities of covered OMIMs genes in the CNVs, by type of CNV found, multivariate analysis such as mean comparison test (Tukey's Multiple test) was applied. A *p-*value less than 0.05 was considered statistically significant.

### Selection and analysis of LCSH

The analysis and selection of LCSH followed the methodology outlined in Chaves & coworkers (2019), applying a threshold of ≥ 3 megabase pairs (Mbp) for the LCSH analysis. This threshold is typically used in clinical investigations, as opposed to population-based studies, where the cut-off threshold is usually considerably lower^24^. All participants who had LCSHs satisfying the above criteria were included, regardless of whether they had or not a pathogenic CNV.

### Automation of LCSHs analyses

For investigation of consanguinity and comparative LCSH analysis among cases as well as for calling potential UPD, all the LCSH reported in ChAS for each case were copied with coded identification and compiled into Excel sheets.

For a more adequate and precise analysis the process was automatized and all LCSHs found in the cohort were imported into Google Colab (https://colab.google/) and manipulated using the Python [3.10] programming language. The libraries used for data manipulation and analysis were Pandas [2.2.0] and NumPy [1.24.0] (for numerical computations). The code used for the analysis is available on the project's GitHub page: https://github.com/tiagochavo87/LCSH_analysis.

### Analysis of consanguinity

The frequency of consanguinity in the cohort was calculated according to Kearney, Kearney and Conlin (2011). In short, when the homozygous patterns suggested inbreeding, all the regions of homozygosity ≥ 3 Mbp distributed throughout the chromosomes were added, with exception of the LCSH located on the sex chromosomes; the total sum in Mbp being divided by the size of the autosomal genome, 2.881 Mbp (GRCh37/hg19). The percentage obtained was correlated with the inbreeding coefficient (F), which is: 25% (first grade; 1/4—parent/child or full siblings), 12.5% (1/8—second grade: half siblings; uncle/niece or aunt/nephew; double first cousins; grandparent/grandchild), 6% (1/16—third grade: first cousins), 3% (1/32 fourth grade: first cousins once removed), 1.5% (1/64—fifth grade: second cousins), < 0.5% (1/128—seventh grade: third cousins)^24^. Kearney and co-workers emphasized that this is a crude calculation, likely to represent an underestimate of the actual homozygous proportion because of the applied threshold of LCSHs over 3 Mbp and because the CMAs may not have SNP probes in certain regions like the acrocentric short arms and the centromeric regions. On the other hand, depending on the degree of inbreeding in the population, these correlations eventually could overestimate the direct kinship relation of the proband.

### Uniparental disomy (UPD)

When only LCSHs 3 to < 5 Mbp were present in the genome, but in one single autosomal chromosome the sum of two or three LCSHs (< 5 Mbp) exceeded 10 Mbp, the homozygous regions were considered a potential isodisomy resulting from a uniparental disomy (UPD) event that underwent previous recombination. When one or more LCSH over 5 Mbp was present in a single chromosome with a size or sum (in the case of multiple LCSHs) ≥ 10 Mbp, it was considered a potential UPD (regardless of eventual LCSHs ≤ 5 Mbp on other chromosomes). If more chromosomes had LCSHs over 5 Mbp, it was not regarded as a potential UPD case^28^.

The ChAS software does not recognize homozygosity, but the absence of heterozygosity named there as loss of heterozygosity (LOH). This includes hemizygous regions generated by a larger deletion. Therefore, all cases with LOHs ≥ 10 Mbp in size on a single autosomal chromosome, regardless of the presence of an additional chromosome with LOH(s) over 10 Mbps in size (or sum of sizes), were manually reviewed, to eliminate the confounding effect of eventual hemizygous regions to call LSCHs and ultimately an UPD.

### Analysis of the most frequent LCSH

Of the 953 files available for LCSH analysis we selected the 917 microarrays for the cytobands that most frequently showed regions with LCSH ≥ 3 Mbp on an autosomal chromosome, and those LCSHs present in more than 5% of individuals were considered common LCSH. This percentage was chosen because the frequency of ≥ 1%, which is the usual threshold to define common polymorphisms of SNPs in a population, was not considered applicable here because this is an affected cohort. Also, others have chosen the same threshold (or lower) to consider LCSH found in an affected cohort as a common variation, likely lacking clinical significance for their analysis^35–39^. Hence, in doing so, we believe to have an adequate safety margin for selecting common LCSH due to ancestral haplotypes rather than due to consanguinity or other pathogenesis-related mechanisms.

To delineate a more accurate genomic position for the most frequent LCSH, the shared homozygous sections were superimposed, and their genomic positions obtained based on the median of their beginning and end.

### Ethics approval and consent to participate

The project was submitted and approved by the Research Ethics Committee of the Hospital Infantil Joana de Gusmão, the children hospital of Florianópolis-SC, Brazil, under the Nr 2339104, and respects the guidelines and criteria established by the resolution 466/12 of the Brazilian National Health Council. Patients or their caregivers signed the Informed Consent Form to participate in the study. In cases in which it was not possible to contact the patient for any justifiable reason (loss of contact information, mainly) the data was used and a Justification of Absence of Consent was signed by the research team, ensuring the commitment to maintain confidentiality and privacy of the patients whose data and/or information was collected in the records.

## Results

Out of the 1012 cases, 615 (61%) were male and 397 (39%) were female, with ages ranging from 0 to 55 years, and a mean age of 10 years (median = 7.15, standard deviation = 10.2).

Previous karyotyping results were available for 182 patients, with 122 normal and 60 abnormal results (for which CMA was requested to identify the specific sequences involved). However, for most patients no information about previous genetic assessments was available.

From the 1012 microarrays, a total of 7150 CNVs which fulfilled the filtering criteria were selected; 3747 duplications and 3403 deletions which were interpreted and classified into benign CNVs, pathogenic CNVs (PCNVs), variants of uncertain clinical significance (VUS) and likely pathogenic CNVs (LPCNVs).

### Phenotypic characterization

Out of the 1012 cases, four were excluded from the phenotypic characterization due to the unavailability of clinical data.

The cohort is mostly characterized by individuals with neurodevelopmental impairment (85%), and 83% of cases had ID and/or DD. In 56% of cases only DD was present while ID was described in 33%. It should be noted that 420 (42%) were under 5 years of age, which is below the age range for intellectual disability diagnosis.

### Phenotypic characterization for cases with ASD

Cases with ASD represent 33% of our cohort, these 333 cases, 77 (23%) were under 5 years of age, below the age for diagnosis of ID, and of these, 17 (22%) had DF. Of the other 256 individuals 5 years or older, 68 had ID, of which 36 also had DF; 43 had only DF, and 145 had "isolated" autism (without ID and dysmorphic features (from Facial dysmorphisms to CAs, see cohort in methodology).

Of the 262 male cases, 59 (53%) were below age 5, the diagnostic age for ID, and of these 12 had DF. Of the 203 male cases, aged 5 or more, 53 presented ID, and of these 29 had DF, whereas 150 (74%) had no ID of which 30 presented DF and 120 presented what we call “isolated” autism.

Of the 71 female ASD cases, 18 (25%) were under age 5, and of these 5 had DF. Of the 53 females aged 5 or more, 15 had ID, and of these 7 also had DF, 38 (72%) had ASD without ID, 13 of them with dysmorphic features (DF) and 25 of them presenting what we call “isolated” autism.

**Figure 1:**
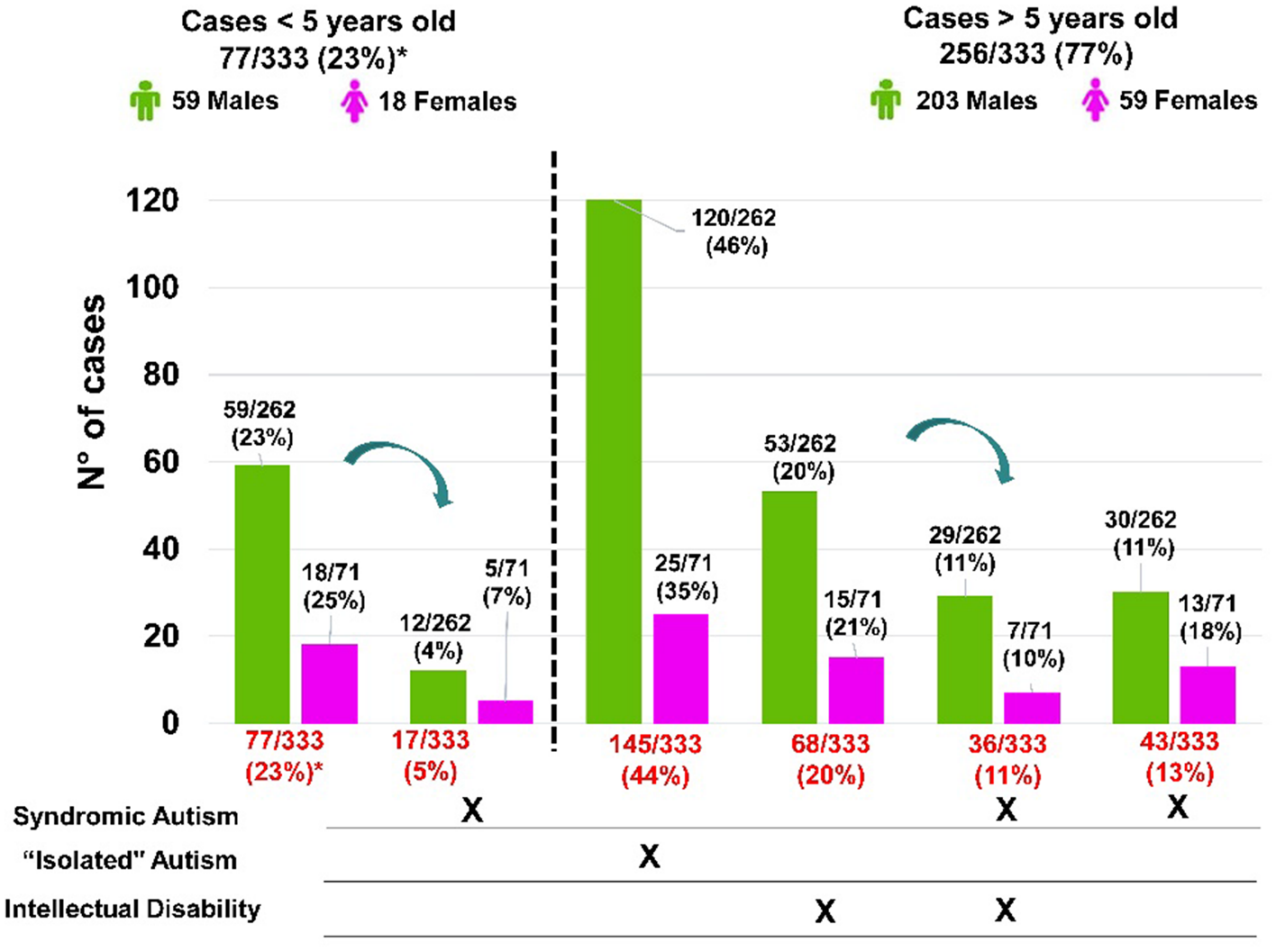
summary of the phenotypic characterization of the cases with ASD.

In Fig. 1 we summarize the phenotypic characterization of the cases that presented ASD in the cohort.

### Other phenotypes

In addition to the main neurodevelopmental phenotypes, most individuals have syndromic features (56%) such as congenital anomalies or malformations or atypical (dysmorphic) facial features (47% of the cohort). Psychiatric or behavioral problems, variations in height or body weight were less frequent accompanying phenotypes.

The phenotypic characteristics recorded in our cohort are listed in Table 1.

**Table 1:** The clinical characteristics recorded for patients with negative (only benign CNVs) and pathogenic (only PCNV) CMA results.

Signs/symptoms	In the cohort (N = 1008)	Negative (N = 706)^a^	Pathogenic (N = 175)^a^	*p*-value	Odds ratio
Characteristics					
Obesity	3% (33)	2% (17)	5% (8)	0.076	0.46
Low weight	5% (55)	2% (34)	9% (16)	0.010*	**0.44**
Abnormal growth	3% (29)	3% (21)	3% (5)	1	1.04
Short stature	10% (104)	9% (67)	14% (23)	0.05	0.60
Slender build	3% (34)	3% (20)	5% (8)	0.233	0.61
Prenatal problems	4% (36)	3% (23)	4% (6)	0.817	0.95
Neurodevelopment	85% (854)	85% (600)	83% (146)	0.639	1.12
Developmental delay	56% (569)	53% (377)	70% (119)	0.0003***	**0.53**
Motor development delay	8% (85)	7% (46)	12% (20)	0.036*	**0.54**
Deafness or hearing loss	3% (31)	3% (19)	4% (7)	0.218	0.58
Speech and language delay and/or dyslalia	21% (216)	21% (151)	26% (44)	0.224	0.79
Difficulty of learning	6% (60)	7% (47)	4% (9)	0.603	1.32
Intellectual disability	33% (330)	31% (216)	41% (69)	0.014*	**0.65**
Mild	4% (37)	3% (24)	2% (4)	–	–
Moderate	2% (16)	2% (11)	2% (4)	–	–
Severe	2% (19)	2% (11)	2% (4)	–	–
Not specified	26% (258)	24% (170)	34% (57)	–	–
Intellectual disability and/or developmental delay	83% (834)	65% (456)	76% (129)	0.025*	**0.65**
Behavioral		–	–		
Behavioral changes (obsessive–compulsive disorder, attention deficit hyperactivity disorder, self and hetero-aggression, behavior disorder, psychosis)	12% (122)	11% (79)	14% (23)	0.509	0.83
Autism spectrum disorder	33% (333)	36% (255)	20% (34)	0.0001****	**2.18**
Congenital malformation(s) and/or dysmorphism(s)	56% (563)	–	–		
Facial malformations/dysmorphisms	47% (471)	43% (305)	65% (110)	0.0001****	**0.42**
Other congenital malformations		–	–		
Musculoskeletal (scoliosis, diaphragmatic hernia, vertebral anomaly)	4% (42)	4% (29)	2% (4)	0.830	1.21
Upper limb anomalies	8% (79)	6% (40)	15% (25)	0.0003***	**0.36**
Lower limb anomalies	8% (83)	6% (45)	15% (25)	0.0015***	**0.41**
Heart anomalies and malformations	8% (79)	7% (48)	12% (20)	0.018*	**0.51**
Gastrointestinal anomalies and malformations	4% (44)	4% (25)	6% (10)	0.1955	0.61
Genitourinary anomalies and malformations	4% (44)	4% (26)	9% (15)	0.004**	**0.38**
Neurologic abnormality	24% (239)	22% (155)	29% (50)	0.071	0.70
Epilepsy	6% (62)	6% (42)	5% (8)	0.856	1.17
Ataxia	2% (18)	1% (10)	2% (4)	0.495	0.61
Hypotonia	7% (70)	7% (51)	8% (14)	0.746	0.90
Abnormal brain structure	11% (112)	10% (72)	14% (24)	0.177	0.71
Seizures	6% (61)	5% (37)	6% (10)	0.850	0.91
Endocrinological abnormalities	4% (39)	3% (21)	5% (8)	0.340	0.64
Cutaneous abnormalities (hyper and hypopigmentation, hemangioma, freckles, café-au-lait spots and others)	3% (29)	2% (16)	4% (7)	0.192	0.56
Hematologic abnormalities	2% (19)	2% (14)	1% (2)	0.751	1.75

^a^Comparison groups diagnosed with pathogenic CNVs (diagnosed) versus the groups without clinically relevant CNVs (no CNVs or only benign CNVs). Cases where VUS and LPCNVs was the most relevant finding (128 individuals) were not considered in the correlation, because they represent inconclusive diagnosis.

*Significant statistical correlation found between pathogenic CNV and phenotype (*p* ≤ 0,05), **p ≤ 0,005, ***p ≤ 0,0005 and ****p ≤ 0,0001.

Significant values are in bold.

### Diagnostic rate and interpretation of CNVs

Within our cohort of 1012 individuals (including 420 previously published cases 33), we identified 358 rare CNVs (VUS, LPCNVs and PCNVs), of which 203 were interpreted as pathogenic and were present in 170 individuals, (including 75 previously published), representing 17% of the cohort. The description of the PCNVs and clinical phenotypes of the carrier patients are listed in Table 2 (without ASD), Table 3 (with ASD), and the previously published are listed in Chaves & coworkers^33^.

**Table 2:** Pathogenic CNVs (PCNV) found in the cohort without ASD.

Case	PCNV	Microarray nomenclature	Size (Kbp)	No. of genes	Some of the relevant genes	Phenotype	Gender/other info	Inheritance	Karyotype	Syndrome
#497	Del	arr[GRCh37] 2p13.2p13.1(72,707,781-73,680,438)x1	973	12	EXOC6B, SPR	Abnormal growth, DD and psychiatric disorder	M/–	ND		–
#501	Del		71,126	401	445 OMIMs	DD, DI, hypoplastic fourth toe, phalangeal malformation	F/ 2 PCNVs	ND	(46,X,der(X)( ~q21-qter)	
		arr[hg19] Xq21.1-q28(84,107,007-155,233,098)x1								
#501	Dup	arr[hg19] 11q13.1q25(65,446,446-134,937,416)x3	69,490	550	729 OMIMs	DD, DI, hypoplastic fourth toe, phalangeal malformation	F/ 2 PCNVs	ND	(46,X,der(X)( ~q21-qter)	
#527	Del	arr[GRCh37] 5p15.33p14.1(113,577-27,590,026)x1	27,476	151	61 OMIMs	Short stature, agenesis of the corpus callo sum, DD, cardiomyopathy and laryngeal web	F/potential UPD5	ND	46,XX,del(5)(~p13-p15)	Cri du Chat¨syndrome
#533	Del	arr[GRCh37] 16q24.2q24.3(87,939,406-89,481,546)x1	1542	38	ANKRD11	Obesity, DD, SLD, ID	F/–	ND	–	KBG syndrome
#542	Dup	arr[GRCh37] 17p12(14,083,055-15,503,234)x3	1420	15	PMP22 (*601097)	Charcot-Marie-Tooth like phenotype	F/–	ND	–	Charcot-Marie-Tooth type 1A—CMT1A
#549	Del	arr[hg19] 8q22.2-q22.3(101,265,736-104,749,739) x1	3484	53	SLC25A3Z (*610815),GRHL2 (*608576)	Gross motor delay	M/–	ND	–	–
#552	Dup	arr[GRCh37] Xq13.2q21.32(71,947,354-93,480,817)x3	21,533	84	49 OMIMs	Fetal loss	F/–	ND	46,XX,dup (X)(q13q22)	–
#568	Del	arr[GRCh37] 3p22.3p22.2(32,654,188-36,570,368)x1	3916	15	TRIM71, CRTAP and GLB1	Hypotonia, poor head support, does not eye track, long face, downslanted palpebral fissures, stridor, glossoptosis, widely-spaced nipples, dolichocephaly, micrognathia hyperconvex toenail, spatulate terminal phalanges, inguinal hernia, macrocephaly, tall stature	F/–	ND	–	–
#574	Del	arr[hg19] Xq28(154,154,958-155,233,731)x1	1079	21	RAB39B (*300774)	Umbilical cord hernia, macrocephaly	F/–	ND	45X,der(X)t(X;15)(q28;q11.2),-15	–
#581	Del	arr[GRCh37] 1p36.33p36.23(849,467-7,883,834)x1	7034	162	GABRD, GLB1 (*611458), CRTAP (*605497)	Newborn, hydrocephalus with mild to moderate colpocephay of the lateral ventricles, w/o intracranial hypertension, microtia grade I, overlapping toes, broad forehead, bulbous nose, in ICU under mechanical ventilation.	F/–	ND	–	¨Chromosome 1p36 Deletion Syndrome
#606	Del	arr[hg19] 5q35.2q35.3 (175,416,095-177,439,550)x1	2023	51	NSD1 (*606681)	Large hands and feet, plagiocephaly, DD, axial hypotonia, dilated Cardiopathy, inguinal hernia	F/–	ND	–	Sotos syndrome
#611	Del	arr[hg19] 1p36.23-p36.33(849,466-2,040,693)x1	2040	66	GABRD (137163), MMP23B (603321)	DD, ID, DF, history of pre-maturity with macrocephaly.	F/–	ND	–	Chromosome 1p36 deletion syndrome
#620	Dup	arr[GRCh37] 15q11.2q13.3(22,770,422-32,915,723)x4	10,145	179	GABRB3(* 137192)	SevID, short stature	F/–	ND	46,XX +mar	Síndrome de Tetrassomia Parcial do 15q
#626	Del	arr[GRCh37] 21q22.12q22.3(37,742,853-42,805,421)x1	5063	49	DYRK1A (600855), KCNJ6 (600877)	DD, microcephaly, abnormal pinna, long phalanges and widely-spaced nipples.	F/–	ND	–	DYRK1A-related intellectual disability syndrome
#632	Del	arr[GRCh37] 22q11.21(18,636,750-21,800,471)x1	3164	91	TBX1 (602054)	quadriparesis with hypotonia, multiple CAs, DD	M/–	ND	–	22q11.2 deletion syndrome
#646	Del	arr[GRCh37] 13q34(114,036,741-115,107,733)x1	1071	22	CHAMP1	Leopard syndrome like frekles, mildID, alopecia, tremor	F/1 VUS	ND	–	–
#651	Del	arr[GRCh37] 5p15.33p15.2(113,577-13,142,487)x1	13,029	103	SLC9A3	DD, ID, seizures, glaucoma	M/ 2 PCNVs	ND	46,XY, add(5)(p15)	Cri du Chat syndrome (#123450)
#651	Dup	arr[GRCh37] 13q31.1q34(87,535,468-115,107,733)x3	27,572	201	81 OMIMs	DD, ID, seizures, glaucoma	M/ 2 PCNVs	ND	46,XY, add(5)(p15)	–
#652	Del	arr[GRCh37] 6q25.3q27(159,427,416-170,914,297)x1	11,487	102	48 OMIMs	microcephaly, DD, ID, FD	F/–	ND	46,XX/46,XX del(6) (q13q15)?	–
#653	Dup	arr[GRCh37] 21q11.2q22.3(15,016,487-48,093,361)x3	10,072	369	167 OMIMs	microcephaly, microtia, DD	M/–	ND	47,XY,del(9)(˜p21-pter),+extra	Partial 21q trisomy
#664	Del	arr[GRCh37] 17p11.2(17,121,644-20,187,953)x1	3066		RAI1 (*607642)	DD, microtia, ventricular septal defect, FD.	M/–	ND	–	Smith-Magenis syndrome
#668	Del	arr[GRCh37] 15q11.2q13.1(23,290,788-28,704,050)x1	5413	126	SNRPN (*182279)	DD, generalized hypotonia, dyslalia, ID, binge eating, motor restlessness, low vision, almond-shaped eyes	F/–	ND	–	Prader Willi syndrome
#676	Del	arr[GRCh37] 6q25.1q27(152,345,416-170,914,297)x1	18,569	146	ARID1B (*614556)	Micrognathia, glossoptosis, agenesis of the corpus callosum, DD	F/–	ND	46,XX del(6)(q2-)	–
#678	Del	arr[GRCh37] 7q11.23(72,701,099-74,141,494)x1	1440	30	ELN (*130160)	DD, hypotonia, bone abnormalities	M/–	ND	–	Williams Beuren syndrome
#687	Del	arr[GRCh37] 5p15.33p15.2(113,577-13,142,487)x1	13,029,	103	CTNND2(*604275)	DD, intraventricular communication, axial polidactilia, FD	F/ 2 PCNVs	ND	46,XX, add(5)(p15)	Cri du Chat syndrome
#687	Dup	arr[GRCh37] 13q31.1q34(87,535,468-115,107,733)x3	27,572	201	CHAMP1	DD, intraventricular communication, axial polidactilia, FD	F/ 2 PCNVs	ND	46,XX, add(5)(p15)	–
#689	Dup	arr[GRCh37] 8p23.3p22(158,049-14,095,397)x3	13,937	152	TNKS1 (*603303), SOX7 (*612202), GATA4 (*600576)	Intraventricular communication, patent foramen ovale, aortic stenosis, pulmonary stenosis, inguinal hernia, long palpebral fissures, DD, ID	F/–	ND	46,XX,14pst+	8p23.3p22 duplication syndrome
#698	Del	arr[GRCh37] 11p14.2p13(26,997,314-33,491,623)x1	6494	47	WT1 (*607102), PAX6 (*607108), BDNF (*113505)	ID, aniridia, cryptorchidism, micropenis, hypogonadism, myopia, FD	M/–	ND	–	WAGR syndrome
#709	Del	arr[GRCh37] 1p36.33p36.22(849,467-11,465,408)x1	10,616	196	GABRD (137163), PRKCZ (176982), SKI (164780)	Syndromic features (not specified)	F/–	ND	–	Chromosome 1p36 deletion syndrome
#712	Del	arr[GRCh37] 2q37.2q37.3(235,913,632-242,782,258)x1	6869	86	HDAC4 (605314), GPR35 (602646)	Sagittal craniostenosis, motor delay, SLD, short fingers, FD	F/–	ND	–	Chromosome 2q37 deletion syndrome
#713	Del	arr[GRCh37] Xq26.2(132,496,732-133,293,329)x0	797	3	GPC4 (300168), GPC3 (300037)	Suspected Beckwith Wiedemann syndrome	M/–	ND	–	Simpsom-Gobali-Behmel syndrome
#720	Del	arr[GRCh37] 12q13.3q14.1(57,075,559-58,481,772)x1	1406	56	KIF5A, MARS	Hypotonia, ModID, psychiatric disorder, elongated face, oblique palpebral fissures, abnormal lip shape	F/–	ND	–	–
#735	Del	arr[GRCh37] 3q22.1q25.2(132,936,742-152,466,305)x1	19,530	147	ZIC4 (*608948), ZIC1 (*600470)	Multiple cas	M/–	ND	–	–
#739	Dup	arr[GRCh37] 8q24.13q24.3(125,496,812-146,295,771)x3	20,799	219	117 OMIMs	DD, ID, interatrial communication, preauricular appendage, FD	F/2 PCNVs	ND	46,XX +mar	–
#739	Dup	arr[GRCh37] 22q11.1q11.21(17,277,402-20,729,389)x3	3452	85	44 OMIMs	DD, ID, interatrial communication, preauricular appendage, FD	F/2 PCNVs	ND	46,XX +mar	Chromosome 22q11.2 microduplication syndrome
#740	Del	arr[hg19] 9q22.33q33.1(102,245,320-119,845,528)x1	17,600	136	ZNF462	Short stature, global DD, microcephaly, FD	M/–	ND	–	–
#746	Del	arr[GRCh37] 1p36.31-p22(9,580,727-11,784,118)x1	2203	38	PIK3CD, MTOR	Failure to thrive, DD, ID, conduct disorder, FD, prominent nasal septum and retrognathia	F/–	ND	–	Chromosome 1p36 deletion syndrome
#760	Del	arr[GRCh37] 12p13.2p12.3(11,867,287-15,360,229)x1	3493	46	GRIN2B (138252)	DD, ID	F/–	ND	–	–
#761	Del	arr[hg19] Xp22.31(6,455,149-8,135,644)x1	1680		STS (*300747)	Growth delay, DD	F/–	ND	–	–
#786	Del	arr[GRCh37] 14q32.33(105,213,585-106,328,827)x1	1115	34	NUDT14 (*609219), BRF1 (*604902), PACS2 (*610423), MTA1 (*603526)	Global DD, hypotonia, SLD, hypotonic face, mesofacial hypoplasia, oblique palpebral fissures, tapered nasal bridge, smooth philtrum	F/–	ND	46,XX add[14](q32.3)	–
#786	Dup	arr[GRCh37] 18q21.31q23(54,211,852-78,013,728)x3	23,802	122	PIGN (*606097)	Global DD, hypotonia, SLD, hypotonic face, mesofacial hypoplasia, oblique palpebral fissures, tapered nasal bridge, smooth philtrum	F/–	ND	46,XX add[14](q32.3)	Partial 18q trissomy
#795	Del	arr[GRCh37] 15q11.2q12(23,214,984-25,778,351)x1	2563	110	14 OMIMs	4 yrs,scoliosis, protruded ears, thin upper lip, tapered fingers, DD	M/–	ND	–	Prader Willi or Angelman syndrome.
#801	Del	arr[GRCh37] Xq22.1q22.3(101,083,092-105,991,325)x1	4908	57	PLP1	FD, hypothyroidism, low weight, DD	F/–	ND	–	–
#807	Dup	arr[GRCh37] Xp22.33p22.13(878,067-19,071,519)x2	18,193	125	90 OMIMs	DD, hypotonia, long hands and feet	M/-	ND	–	–
#811	Dup	arr[GRCh37] 12p13.33p12.1(173,787-21,971,638)x3	21,798	312	217 OMIMs	FD, strabismus, bulbous nose, epicanthus, dysphagia	M/-	ND	46,XY,add(18)(~p11.32)	–
#825	Del	arr[GRCh37] 2q31.1q31.3(175,599,084-181,995,668)x1	6397	57	HOXD10 (*142984)	ASD, camptodactily of 2^nd^ fingers, deformity of 5th fingers, DD, dyslalia	M/-	ND	–	Chromosome 2q31.2 microdeletion syndrome
#826	Del	arr[GRCh37] 20q13.13(47,150,102-49,526,051)x1	2376	38	ADNP, KCNB1	SLD, congenital cardiopathy, nephropathy, cognitive delay and motor delay	F/–	ND	–	–
#828	Dup	arr[GRCh37] 1q21.1q21.2(146,106,724-147,830,830)x3	1724	24	GJA5 (121013), GJA8 (600897)	ID, strabismus and protruding ears Brother of case #829	M/ 1 VUS	ND	–	Chromosome 1q21.1 duplication syndrome
#829	Dup	arr[GRCh37] 1q21.1q21.2(146,106,724-147,830,830)x3	1724	24	GJA5 (121013), GJA8 (600897)	ID, strabismus and protruding ears. Brother of case #828	M/ 1 VUS	ND	–	Chromosome 1q21.1 duplication syndrome
#848	Del	arr[GRCh37] 16p11.2(29,591,327-30,190,029)x1	599	22	32 OMIMs	Temporal lobe epilepsy, in clusters.	F/-	ND	–	Chromosome 16p.11.2 deletion syndrome
#861	Dup	arr[GRCh37] 8p23.3p21.1(158,049-27,502,930)x3 pat	27,345	263	129 OMIMs	Low weight, elongated face, synophrys and pointed fingers	M/ 2 PCNVs	Father with partial trisomy of chr 8 and monosomy of chr 12 karyotype	–	–
#861	Del	arr[GRCh37] 12p13.33p13.31(173,787-5,952,112)x1 pat	5778	65	40 OMIMs	Low weight, elongated face, synophrys and pointed fingers	M/ 2 PCNVs	Father with partial trisomy of chr 8 and monosomy of chr 12 karyotype	–	–
#862	Dup	arr[GRCh37] Xp11.23p11.22(48,224,463-52,841,006)x3	4617	102	78 OMIMs	FD, camptodactyly, nasal voice, SLD, ID e finger-like thumb	F/–	ND	–	Chromosome Xp11.23p11.22 duplication syndrome
#875	Del	arr[hg19] 4q28.3q31.21(136,216,198-14,1932,587)x1	5716	–	MAML3-608991	Failure to thrive, DD, SLD, ADHD, hypermetropia	F/–	ND	–	–
#927	Del	arr[GRCh37] 15q11.2q13.1(23,620,192-28,704,050)x1	5084	120	UBE3A (*601623)	Suspected Angelman syndrome	M/ 2 VUS	ND	–	Angelman syndrome
#944	Del	arr[GRCh37] 7q11.23(72,692,113-74,136,633)x1	1445	30	ELN (*130160)	Suspected Williams-Beuren syndrome. Father with tuberous sclerosis	M/–	ND	–	Williams Beuren syndrome
#949	Del	arr[GRCh37] 2p14(64,103,858-67,815,028)x1	3711	34	SLC1A4, SPRED2	Suspected genetic condition	M/–	ND	–	2p14 microdeletion syndrome
#953	Del	arr[GRCh37] 6p25.3p25.2 (156974-4009868)x1	3853	41	FOXC1, RIPK1	Behavioural issues, FD, altered dentinogenesis, low weight, low height	M/–	ND	45,XX,der(6)t(6;14)(p25;q11.2) – 14	6p25 deletion syndrome
#960	Del	arr[GRCh37] 17q21.31(43,710,150-44,214,816)x1	505	10	KANSL1(*612852).	DD, SLD, dyslalia, ID, global hypotonia, no sphincter control	F/–	ND	–	Koolen de Vries syndrome
#961	Dup	arr[GRCh37] 12p13.31p13.33(1,73,786-34,759,042)x3	33,639	397	–	DF, cleft palate, anal fistula, imperforate anus, hypertrophic cardiomyopathy, hypodense area affecting the cortical region, pigmentary lesion in the right eye.	M/–	ND	–	Trisomy of the Chromosome 12p ¨
#985	Del	arr[GRCh37] 15q24.1q24.2 (72,965,465-76,073,450)x1	3108	70	SIN3A	Prognathism, clinodactyly, DD, partial syndactyly	F/–	ND	–	Chromosome 15q24 Microdeletion syndrome
#993	Del	arr[GRCh37] 7q11.23(72,723,370-74,136,633)x1	1413	30	ELN	DD, ligamentous laxity, tricuspid reflux, pulmonary stenosis.	F/–	ND	–	Williams Beuren syndrome
#995	Del	arr[hg19]8p23.1-8p23.3(158,048-6,999,114)x1	6886	48	ARHGEF10	Failure to thrive, FD, hydrocephalus, thin corpus callosum, ventricular ectasia.	F/ 3 PCNVs	ND	46,XX,add(8)(?-pter)	Recombinant chromosome 8 syndrome or San Luis Valle syndrome
#995	Dup	arr[hg19]8p12-p23.1 (11,935023-31,833,216)x3	29,487	181	–	Failure to thrive, FD, hydrocephalus, thin corpus callosum, ventricular ectasia.	F/ 3 PCNVs	ND	46,XX,add(8)(?-pter)	Recombinant chromosome 8 syndrome or San Luis Valle syndrome
#995	Dup	arr[hg19] 8q24.3 (144,794,838-146,295,771)x3	1501	77	–	Failure to thrive, FD, hydrocephalus, thin corpus callosum, ventricular ectasia.	F/ 3 PCNVs	ND	46,XX,add(8)(?-pter)	St. Louis Valley syndrome
#1005	Del	arr[hg19] 16p11.2 (29,580,020-30,190,029)x1	610	32	ALDOA (*103850)	DD, SLD, FD	F/–	ND	–	Chromosome 16p.11.2 deletion syndrome
#1025	Dup	arr[GRCh37] 4q31.23q35.2(149,377,750-190,957,460)x3	41,580	210	110 OMIMs	FD, deafness	F/–	ND	46,XX add(4) ~ q35	Chromosome 4q31.23q35.2 trisomy
#1033	Dup	arr[hg19] 22q11.21 (18,916,842-21,461,017)x3	2544	79	TBX1(*602054)	DD, SLD, ID, seizures and ADHD	M/–	ND	–	22q11.21 duplication syndrome
#1034	Del	arr[hg19] 13q22.3(78,451,099-78,483,275)x1	32	2	EDNRB (*131244)	Deafness, iris heterochromia, white lock. Sister of #1035	F/–	ND	–	Waardenburg syndrome type 4A
#1035	Del	arr[hg19] 13q22.3(78,451,099-78,483,275)x1	32	2	EDNRB (*131244)	Deafness, iris heterochromia, white lock Sister of #1034	F/–	ND	–	Waardenburg syndrome type 4A
#1042	Del	arr[hg19] 22q11.21 (18,648,855-21,800,471)x1	3151	97	TBX1(*602054)	ataxia, DD, FD, epicanthus and oblique palpebral fissures	M/–	ND	–	Di George syndrome
#1047	Del	r[hg19] 2q37.1q37.3(235,387,296-242,782,258)x1	7395	89	HDAC4 (*605314)	DD, motor delay, ligamentous laxity	F/-	ND	-	Chromosome 2q37 deletion syndrome
#1048	Del	arr[hg19] 13q22.1q31.1(74,541,519-81,906,132)X1	7365	48	EDNRB (*131244), SPRY2 (*602466)	DD, motor delay, ID, FD, pectus excavatum	F/–	ND	–	–
#1059	Del	arr[hg19] 5q22.1q23.3 (110,061,210-128,612,286)x1	18,551	104	APC (*611731)	Valvular and supravalvular stenosis, single kidney on the right, failure to thrive	F/–	ND	–	–
#1068	Del	arr[GRCh37] 15q11.2(22,770,422-23,277,436)x1	507	7	TUBGCP5 (*608147), CYFIP1 (*606322), NIPA2 (*608146), NIPA1 (*608145)	Failure to thrive, microcephaly, DD, SLD, ID, psychomotor agitation, ASD, congenital cardiopathy, bilateral ectopic testis, right hemiparesis, altered MRI	M/–	ND	–	Chromosome 15q11.2 deletion syndrome
#1074	Dup	arr[GRCh37] 7q31.32q33(122,745,868-136,171,005)x3	13,425	125	LEP (*164160)	DD, SLD, ID, FD, enlarged thumbs, hirsutism (legs, arms, back), short stature, microcephaly	F/–	ND	–	7q31.32q33 partial trisomy
#1080	Dup	arr[GRCh37] 10p15.3p13(100,048-14,753,970)x3	14,654	124	53 OMIMs	DD, speaks few words, FD, seizures agitation, lack of concentration, oblique palpebral fissures, flat feet, brachydactyly, vitiligo(?) mother has a translocation	F/-	ND	–	–
#1080	Del	arr[GRCh37] 15q26.3(100,995,007-102,429,040)x1	1434	22	10 OMINs	DD, speaks few words , FD, seizures agitation, lack of concentration, oblique palpebral fissures, flat feet, brachydactyly, vitiligo(?) mother has a translocation	F/–	ND	–	–
#1080	Dup	arr[GRCh37] 10p15.3p13(100,048_14,753,970)x3	14,654	124	53 OMIMs	DD, speaks few words, DF, seizures, restlessness and lack of concentration, oblique palpebral fissures, flat feet, brachydactyly, investigation of vitiligo. Mother has a translocation	F/–	ND	–	–
#1083	Del	arr[GRCh37] 8q23.1q24.11(109,456,954-118,350,705)x1	8894	25	TRPS1(*604386)	Suspected trichorhinophalangeal syndrome.	F/–	ND	–	Trichorhinophalangeal syndrome (TRPS).
#1109	Dup	arr[GRCh37] 4q31.21q35.2(141,800,494-190,957,460)x3	49,157	254	136 OMIMs	Test was made to identify additional material on chromosome 3	M/ 2 VUS	ND	Mentions Karyotype with additional material on chromosome 3	–
#1113	Del	arr[GRCh37] 22q11.21(20,716,877-21,800,471)x1	1084	31	LZTR1 (600574), SERPIND1 (142360)	Discrete DD, dyslalia, microphthalmia, bilateral leucoma, bilateral exophthalmos, oblique palpebral fissures, telecanthus, limitations in elbow extension, left equinovarus foot	F/–	ND	–	Distal 22q11.2 deletion syndrome
#1117	Del	arr[hg19] 13q14.13q21.33(47,060,617-72,679,280)x1	25,619	124	51 OMIMs	Motor delay, dolichocephaly, high palate, strabismus	M/–	ND	46,XY,del(13)(q22q31)	Chromosome 13q14 deletion syndrome

Does not include cases previously published in^33^.

Pathogenic CNVs (PCNVS) found by CMA in the cohort, with the number of genes present in the region, listing some the relevant genes and available phenotypes for each individual.

Under column gender/“other info”: patient may have 2 PCNVs, or additionally 1 VUS or 1 LPCNVs. All PCNVs are listed in this table. VUS and LPCNVs are listed in another table.

*Dup* duplication, *Del* deletion, *CAs* congenital anomaly, *DD* developmental delay, *mildID* mild intellectual disability, *ModID* moderate intellectual disability, *SevID* severe intellectual disability, *ASD* autism spectrum disorder,* FD* facial dysmorphism, *SLD* speech and/or language delay or impairment, *IUGR* intrauterine growth restriction, *ADHD* attention-deficit/hyperactivity disorder, *LD* learning difficulty, *ND* not determined, *F* female, *M* male, *VUS* CNV of uncertain significance, *LPCNVs* likely pathogenic CNVs.

**Table 3:** Pathogenic CNVs found in the ASD Cohort. Includes ASD cases of the cohort previously published^33^.

Case	PCNV	Microarray nomenclature	Size (Kbp)	No. of genes	Some of the relevant genes	Phenotype	Gender/other info	Inheritance	Karyotype	Syndrome
#15	Del	arr[hg19] 16p11.2(28,689,085–29,043,863)x1	355	18	SH2B1	DD, ASD	M/affected brother (#16)	ND		Distal 16p11.2 deletion syndrome
#16	Del	arr[hg19] 16p11.2(28,689,085–29,388,495)x1	362	18	SH2B1	DD, ASD	M/affected brother (#15)	ND		Distal 16p11.2 deletion syndrome
#52	Del	arr[hg19] 22q13.33(50,788,193–51,115,526)x1	327	18	SHANK3	SevID, ASD, motor difficulties, FD, CAs and epilepsy	M/	ND		Phelan-McDermid syndrome
#66	Dup	arr[hg19] 15q25.1q26.3(80,304,866–102,429,040)x3	22,124	175	IGFR1, AKAP13, CPEB1, NTRK3, WDR73	SevID, ASD, convulsions, SLD, hyperactivity, CAs (one kidney) and FD	M/–	ND		–
#69	Del	arr[hg19] 16p12.2p11.2(21,405,327–29,388,495)x1	7983	82	SH2B1	MildID, ASD, SLD, hyperactivity and FD	M/–	ND		–
#70	Dup	arr[hg19] 7q11.23(72,732,834–74,155,067)x3	1422	27	WBSCR27, WBSCR28	ModID, ASD and hyperactivity	M/–	ND		Williams-Beuren region duplication syndrome
#76	Dup	arr[hg19] 7q11.23(72,556,215–74,245,599)x3	1689	34	WBSCR27, WBSCR28	MildID, ASD	M/ -	ND		Williams-Beuren region duplication syndrome
#77	Del	arr[hg19] 15q13.2q13.3(31,073,735–32,446,830)x1	1373	9	CHNA7	MildID, ASD and hyperactivity	M/–	ND		–
#148	Dup	arr[hg19] Xp22.3q28(1–247,249,719)x3 ou arr(X)x3	155,270	–	–	DD, ASD and schizophrenia	F/–	ND		Triple X syndrome
#184	Del	arr[hg19] 15q11.2q13.1(22,770,421–28,823,722)x1	6053	121	UBE3A, SNRPN	DD, ID, epilepsy, ASD and ADHD	M/–	ND		Angelman syndrome
#235	Dup	arr[hg19] 17p11.2(16,591,260–20,473,937)x3	3882	68	RAI	Slender build, DD, SLD, ModID, ASD and FD	F/–	ND		Potocki-Lupski syndrome
#255	Dup	arr[hg19] 22q11.21q11.23(18,493,187–24,313,652)x3	5820	125	TBX1	DD, ASD and FD	M/–	ND		22q11.21 Duplication Syndrome
#345	Del	arr[hg19] 14q32.2q32.31(100,095,248–102,755,064)x1	2660	117	PEGS (DLK1 and RTL1), MEGS (MEG3 and MEG8)	Low weight, short stature, prematurity, IUGR, ataxia, scoliosis, DD, SLD, SevID, ASD, FD and early puberty	F/–	ND		Temple Syndrome
#385	Del	arr[hg19] 21q22.12q22.2(35,834,713–39,831,660)x1	3997	32	DYRK1A	Convulsions, DD, ID, SevID, ASD, cardiomyopathy, CAs (abnormal external genitalia) and thrombocytopenia	M/–	ND		21q22.12 Microdeletion Syndrome
#416	Del	arr[hg19] 18q21.32q23(58,921,746–78,013,728)x1	19,092	75	PIGN	Obesity, CASs, DD, ID, deafness, ASD, FD, and thrombocytopenia	M/–	ND		18 q21.32-qter deletion syndrome
#443	Dup	arr[hg19] 22q12.3q13.1(35,888,588–38,692,765)x4	2804	59	45 OMIMs	Low weight, short stature, DD, SLD, ASD, behavioral disorder DF and mongolian spots	M/–	ND		–
#455	Dup	arr[hg19] Yp11.31p11.2-Yq11.23(2,650,140–28,799,937)x2	26,149	486	39 OMIMs	ASD and tall stature	M/–	ND	47, XY, + mar	XYY-region syndrome
#470	Del	arr[hg19] 2q37.3(238,092,121–242,782,258)x1	4690	73	HDAC4	Asperger's syndrome	F/–	ND		2q37.3 microdeletion syndrome
#511	Dup	arr[hg19] 2q11.2(99,222,915–101,919,539)x3	2696	29	13 OMIMs	ASD, ID, tall stature, CAs and FD	M/1 of 2 pCNVs	ND	47,XY + mar(64%)/48,XY, + + mar(6%)	–
#511	Dup	arr[hg19] 2q11.1q11.2(95,327,873–98,719,140)x4	3391	52	24 OMIMs	ASD, ID, tall stature, CAs and FD	M//1 of 2 pCNVs	ND	47,XY + mar(64%)/48,XY, + + mar(6%)	–
#586	Del	arr[hg19] 15q21.3(57,289,688–57,510,425)x1	221	1	TCF12	ASD, hyperactivity and FD (Asymmetric facies)	M/-	ND		–
#594	Dup	arr[hg19] 1q32.3q41-1q43q44(212,011,806–249,181,598)x3	36,743	581	169 OMIMs	ASD, ID, CAs and FD	F/1 of 2 pCNVs	ND	46,XX, add(22)(q13)	1q32.3-qterm trisomy
#594	Del	arr[hg19] 22q13.31q13.33(47,771,299–51,197,766)x1	3426	49	29 OMIMs	ASD, ID, CAs and FD	F/1 of 2 pCNVs	ND	46,XX, add(22)(q13)	Phelan-Mcdermid syndrome
#667	Del	arr[hg19] 18q12.3(42,453,211–42,988,420)x1	535	3	SETBP1 ,SLC14A2	ASD	M/–	ND		Intellectual developmental disorder, autosomal dominant 29
#714	Del	arr[hg19] 16p11.2(29,591,326–30,190,029)x1	598	31	20 OMIMs	Asperger's syndrome	M/–	ND		Chromosome 16p.11.2 deletion syndrome
#737	Dup	arr[hg19] 16p13.3p12.3(85,880–18,242,713)x3	18,156	342	CREBBP	ASD, FD and CAs	F/1 of 2 pCNVs	ND		Partial trisomy 16p13.3 syndrome
#737	Del	arr[hg19] Xq27.3q28(145,443,311–155,233,098)x1	9723	167	FMR1, AFF2	ASD, FD and CAs	F/1 of 2 pCNVs	ND		–
#751	Del	arr[hg19] 18q12.2q21.1(36,210,635–44,530,609)x1	8319	28	SETBP1	ASD, FD and CAs	M/-	ND		18q deletion syndrome
#791	Del	arr[hg19] 14q12(29,197,241–29,514,397)x1	317	4	FOXG1	ASD, DD, SLD, FD, CAs and seizures	F/–	ND		FOXG1 syndrome
#809	Dup	arr[hg19] Xq28(153,123,879–153,621,056)x2	497	21	MECP2	ASD and CAs	M/–	ND		MECP2 duplication syndrome
#853	Del	arr[hg19] 16p11.2(29,591,326–30,176,508)x1	585	31	20 OMIMs	ASD, SLD, FD, dyslalias and motor difficulties	M/–	ND		Chromosome 16p.11.2 deletion syndrome
#873	Del	arr[hg19] 13q33.2q34(105,020,842–115,107,733)x1	10,086	86	EFNB2, LIG4, SOX1	ASD, ID, CAs, FD and microcephaly	F/–	ND		Distal 13q deletion syndrome
#913	Dup	arr[hg19] 15q24.1q24.2(72,899,646–75,567,198)x3	2667	52	32 OMIMs	ASD, FD and CAs	M/–	ND		–
#970	Dup	arr[hg19] 1q21.1q21.2(146,106,723–147,830,830)x3	1724	56	SATB2	ASD	F/-	ND		1q21.1 microduplication syndrome
#1026	Dup	arr[hg19] 2q33.1(200,182,545–201,185,809)x3	1003	8	SATB2	ASD, ID and DF	M/ potencial UPD: 22q13.1q13.33 (13.2 Mbp; 37,977,281–51,157,531)	ND		–
#1050	Del	arr[hg19] 9p24.3p24.1(208,454–5,222,238)x1	5013	27	DMRT1, DMRT2, DMRT3	ASD, ID, pectus excavatum and FD	M/–	ND		–
#1100	Del	arr[hg19] 15q13.2q13.3(31,098,690–32,444,261)x1	1346	18	CHRNA7	Asperger's syndrome	F/–	ND		15q13.3 microdeletion syndrome
#1107	Del	arr[hg19] 9p24.3p22.3(208,454–15,424,987)x1	15,216	137	NFIB, FREM1	ASD	M/–	ND	46, XY, del(9)(~ p22.2-pter)	9p deletion syndrome

Pathogenic CNVs found by CMA in the cohort with ASD, with the number of genes present in the region, listing the most relevant genes and phenotypes for each individual.

*Dup* duplication, *Del* deletion, *CAs* congenital anomaly, *DD* developmental delay, *MildID* mild intellectual disability, *ModID* moderate intellectual disability, *SevID* severe intellectual disability, *ASD*  autism spectrum disorder, *FD* facial dysmorphism, *SLD* speech and/or language delay or impairment, *IUGR* intrauterine growth restriction, *ADHD* attention-deficit/hyperactivity disorder, *LDO* learning difficulty only, *LD* learning disability, *ND* not determined, *F* female, *M* male, *1 of 2 pCNVs* 1 of 2 patogenic CNVs from one individual.

Among the 170 individuals with pathogenic CNVs of the whole cohort of 1012 cases, including those previously published by Chaves & coworkers^33^, 26 carried more than one PCNV. 19 of them were carriers of 2 PCNVs (cases #33, #47, #61, #127, #251, #331, #332, #372, #407, #501, #511, #594, #651, #687, #737, #739, #786, #861, and #1080). Additionally, seven cases had three pathogenic CNVs (cases #151, #188, #196, #219, #270, #392, and #995). In three cases (#81, #255, and #331), a pathogenic CNV was accompanied by VUS.

Out of the 204 pathogenic CNVs, 119 were deletions, resulting in only one copy of the involved sequence, except for case #713. The deletion in this case involved a genomic region of the boy's single X sex chromosome. And six cases (#81, #255, #331, #646, #927 and #1109), along with a pathogenic deletion, also presented VUS.

**Figure 2:**
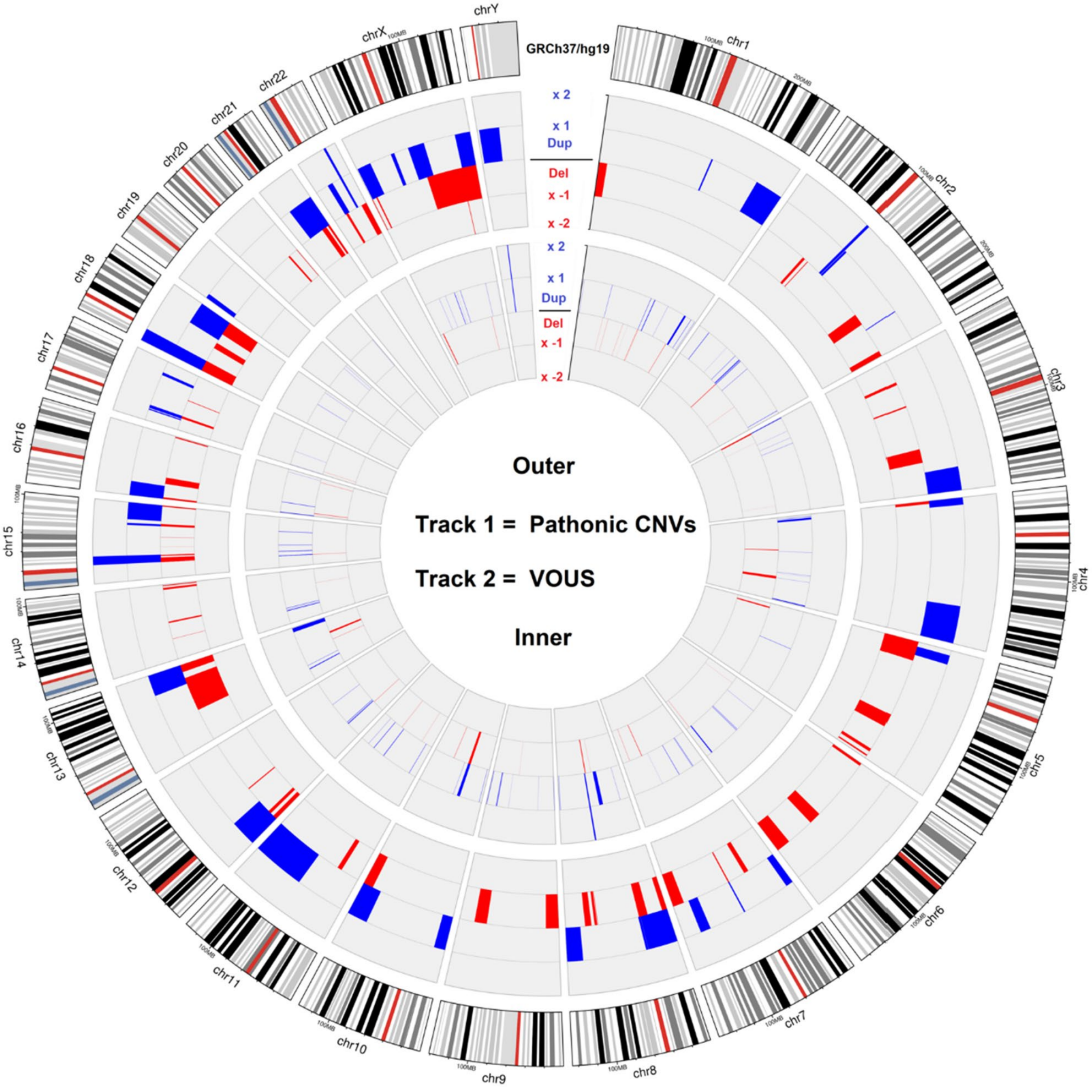
Circle plot with the pathogenic CNVs and VUS* detected in our study.

The other 74 pathogenic CNVs were duplications, which usually result in a total of three copies of the involved sequence, but in eight males (#24, #25, #116, #151, #30, #455, #807 and #809) involved a relevant region of a sex chromosome and resulted in two copies (the main reason for pathogenicity is the fact that in males none of the duplicated copies on X undergoes inactivation, which it does in females) and in five cases (#306, #422, #443, #511 and #620) the CNV found was in a state of four copies. Figure 2 illustrates the frequency and number of pathogenic CNVs found per chromosome.

Pathogenic CNVs were found on all chromosomes (see supplementary information 1—Pathogenic CNVs per chromosome), with sizes from 32 Kbp to 71 Mbp (SD = 9992, mean = 8365) and contained 1 to 581 genes per PCNV (SD = 93, mean = 87), of which 1 to 87 (SD = 13, mean = 9) are genes cited in the OMIM database (OMIM genes) (see supplementary information 2).

Univariate analysis (Fisher's test) indicated the predictive phenotypes for a higher diagnostic outcome (greater chance of having a pathogenic CNV) in our cohort with DNNs: Developmental delay (*p*-value ≤ 0.001, OR = 0.53); Autism Spectrum Disorder (*p*-value ≤ 0.001, OR = 2.18); Facial Malformations/Dysmorphisms (*p*-value ≤ 0.001, OR = 0.42); Upper limb anomalies (*p*-value ≤ 0.001, OR = 0.36); Lower limb anomalies (*p*-value = 0.001, OR = 0.41); genitourinary anomalies and malformations (*p*-value = 0.004, OR = 0.38); Low weight (*p*-value = 0.01, OR = 0.44); Intellectual disability (*p*-value = 0.014, OR = 0.65); Heart anomalies and malformations (*p*-value = 0.018, OR = 0.51); ID or DD (*p*-value = 0.025, OR = 0.65) and Motor development delay (*p*-value = 0.036, OR = 0.54). There was no significantly higher diagnostic result by CMA for the other phenotypes (see supplementary information 3).

Following the scoring system, another 155 rare CNVs were interpreted as 141 Variants of uncertain significance (VUS) (Supplementary Table 1) and 14 as Likely Pathogenic CNVs (LPCNVs) (Table 4), these being the main findings in 13% of the cohort. Of these, 102 are duplications and 53 are deletions. In cases #635, #658, #929 2 VUS were detected and in cases #649, #937, 3 VUS.

**Table 4:** Likely pathogenic CNVs found in the cohort.

Case	CNV	Microarray nomenclature	Size (Kbp)	No. of genes	No. of genes in OMIM	Important genes	Phenotype	Gender/notes
#1015	Dup	arr[hg19] 1q21.3(153,568,824–154,833,332)x3	1264	39	26	GATAD2B	ASD, ID and obesity	F/–
#1127	Del	arr[hg19]2q31.2(179,396,924–179,629,278)x1	232	2	2	TTN (*188840)	ASD, epilepsy	M/–
#513	Dup	arr[hg19] 10q11.22q11.23(46,252,072–51,903,756)x3	5652	61	–	–	ASD and ID	F/–
#519	Del	arr[GRCh37] 9q21.2(79,995,119–80,139,559)x1	144	3	2	VPS13A (605978), GNA14 (604397)	MildID, ADHD	F/–
#547	Dup	arr[GRCh37] 8q12.1q12.3(56,379,919–63,866,456)x3	7487	43	21	CHD7 (*611238)	ptosis, extrahepatic portosystemic shunt type Ib, patent foramen ovale, left ventricular hypertrophy	M/–
#596	Del	arr[hg19] 8q22.2 (100,067,471–100,622,400)x1	555	3		VPS13B (*607817)	DD, obesity, ID, anxiety,diabetes mellitus	F/–
#597	Del	arr[GRCh37] 12p11.23(27,316,348–27,796,495)x1	480	7	3	PFFIBP1 (*603141)	recurrent otitis, seizure, precocious puberty, SLD, broad forehead, long eyelashes	F/
#633	Del	arr[hg19] 6q26(162,374,660–162,738,968)x1	364	1	1	PARK2	ASD	M/
#823	Del	arr[hg19] 5q34q35.1(165,498,746–169,954,911)x1	4456	42	29	KCNMB1 (603951)	DD, speech disorder, short frenulum, low weight, short stature, FD, speech delay, consanguineous parents	F/–
#829	Del	arr[GRCh37] 5p15.31p15.2(9,090,338–11,635,988)x1	2545	20	8	–	ID, strabismus, protruding ears brother of case #828	M/ + 1 PCNVCNV
#833	Del	arr[hg19] 1q21.1(145,252,423–145,888,926)x1	637	24	12	–	ASD	M/–
#847	Del	arr[hg19] 1p12p11.2(120,527,347–120,617,367)x1	90	1	1	NOTCH2	ASD and microcephaly	M/–
#852	Del	arr[hg19] 2q13(110,498,141–110,980,295)x1	482	11	3	NPHP1 (*607100)	Auditory processing disorder, LD, microcephaly	M/–
#956	Del	arr[hg19] 14q22.1q22.2(52,412,733–54,387,154)x1	1974	14	4	ACTR2, Rab-1A	Low weight, short stature, broad forehead, triangular face, everted lips, ogival palate, congenital cardiopathy, SLD	F/–

Likely pathogenic CNVs (LPCNVs), found in the cohort, with the number of genes present in the region, listing some of the relevant genes and available phenotypes for each case.

*Dup* duplication, *Del*  deletion, *CAs* congenital anomalies, *DD* developmental delay, *ID* non-specified intellectual disability, *mildID* mild intellectual disability, *ModID* moderate intellectual disability, *SevID* severe intellectual disability, *ASD* autism spectrum disorder, *FD* facial dysmorphisms, *SLD *speech and/or language delay/impairment, *IUGR* intrauterine growth restriction, *ADHD* attention-deficit/hyperactivity disorder, *LD *learning difficulty, *ASD* autism spectrum disorder, *F* female, *M* male.

These variants were found on most chromosomes except for 21 and 22 (see supplementary information 1—VUS per chromosome), with sizes from 30 Kbp to 8 Mbp (SD = 1266, mean = 802) and contained 1 to 87 genes (SD = 13, mean = 9), of which 1 to 38 (SD = 5 mean = 5) are genes cited in the OMIM database (OMIM genes) (see supplementary information 2). Figure 2 illustrates the frequency and amount of VUS per chromosome (in track 2). Fourteen VUS, according to the scoring system were found to be LPCNVs (Table 4).

All other CNVs were interpreted as either common genetic polymorphisms or benign variants found in all chromosomes, with sizes that varied from 10 Kbp to 24 Mbp (SD = 586, mean = 298) and contained zero to 227 genes (SD = 8, mean = 3), of which zero to 144 (SD = 4 mean = 1) are genes cited in the OMIM database (OMIM genes) (see supplementary information 2).

### Diagnostic rate and interpretation of CNVs for cases with ASD

**Figure 3:**
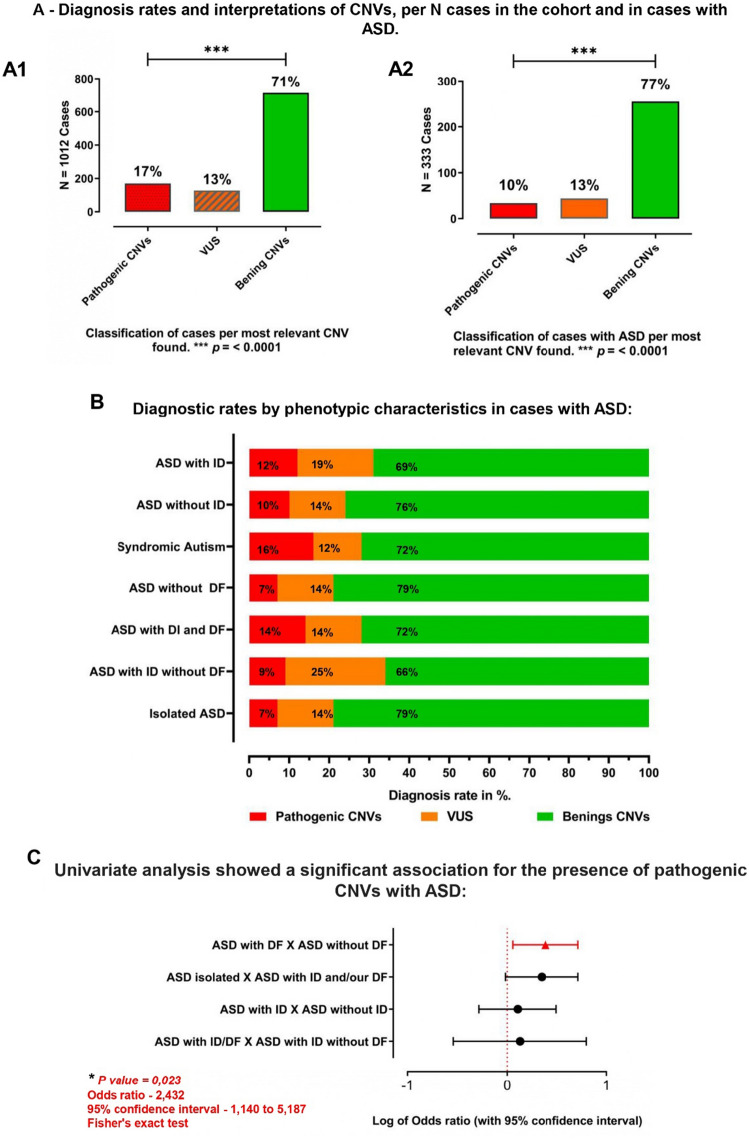
(**A1**) Classification of cases per most relevant CNV found in the whole cohort. (**A2**) Classification of cases per most relevant CNV found in the sub-cohort with ASD. (**B**) Diagnostic rates per ASD phenotypic categories. *ASD* autism spectrum disorder, *ID* intellectual disability, *DF* dysmorphic features (syndromic), classical autism (including ASD cases high functioning isolated ASD), isolated ASD: ASD without ID and without DF/CAs. (**C**) Odds ratios for pathogenic CNVs in classes of phenotypes. Odds ratios shown in log2 scale. As can be seen in (**B**), when comparing ASD with ID to ASD without ID, the diagnostic rate (12% and 10% respectively of PCNVs) is a little higher when ID is present. However, the presence of VUS is 5% higher when ID is present (19% compared to 14% in ASD w/o ID). Syndromic ASD definitively has a much higher diagnostic rate (16%) than non-syndomic ASD (7%).

When analyzing separately the 333 CMAs from patients where ASD (including all definitions of the spectrum) was cited as the main reason for referral or as one of several phenotypes of the patient, a total of 3259 CNVs that met the filtering criteria were detected. Of those 1494 were duplications and were 1765 deletions, most of them interpreted as benign. In 33 CMAs no CNVs meeting the filtering criteria were detected. The frequency of the most relevant type of CNV found in each case in the whole cohort and the sub-cohort with ASD is illustrated in Fig. 3A1, A2. The proportional contribution of each type of CNV per subclass of ASD is illustrated in Fig. 3B.

In 10% of cases (33/333) we identified a total of 38 rare CNVs that were interpreted as pathogenic (Table 3), 22 deletions and 16 duplications. The particularities of cases #511, #594 and #737, with 2 PCNVs, cases #455 (Y Chromosome), #809 (X chromosome) and cases #443 and #511 (PCNV in a four-copy state) were mentioned before.

In the ASD sub-cohort pathogenic CNVs were found on 14 of the 24 human chromosomes (1, 2, 7, 9, 13, 14, 15, 16, 17, 18, 21, 22, X and Y), with sizes from 221 Kbp to 22 Mbp (SD = 5561, mean = 4926) and contained 1 to 342 genes (SD = 63, mean = 60), of which 1 to 83 (SD = 32, mean = 29) are genes cited in the OMIM database (genes OMIM) (see supplementary information 3).

For individuals affected with syndromic ASD (with DF) the diagnostic rate was higher than for the whole ASD cohort (16% to 10%), confirmed by univariate analysis 16% (p = 0.02, OR 2.43, for pathogenic CNVs) (Fig. 3C).

In cases with ASD, DF and ID, the diagnostic rate was 14%, and for ASD with ID, but without DF, it was 12%. For "isolated" ASD, the diagnosis dropped to 7%.

In the 39 cases < 5 years, 5 (13%) had pathogenic CNVs and 6 had only VUS.

For 13% (44/333) of the cases, VUS, which are also rare CNVs, were the only relevant findings, totaling 48 CNVs, 20 deletions and 28 duplications (Supplementary Table 1). These variants also were found on most chromosomes, except for chromosomes 4, 5, 12, 18, 19, 20, 21 and 22, with sizes from 10 Kbp to 5.6 Gbp (SD = 1032 Kbp, mean = 700 Kbp) and contained 1 to 61 genes (SD = 12, mean = 9), of which 1 to 26 (SD = 5 mean = 4) are genes cited in the OMIM database (OMIM genes) (see supplementary information 3). In tracks 3 and 4 of the circus ideogram graph (see supplementary information 4), the VUS found per chromosome are plotted.

Four of these VUS (in cases #513, #633, #833 and #1127) were subclassified as LPCNVs, currently without convincing evidence (Table 4).

All other CNVs were interpreted as either benign or common genetic polymorphisms, submicroscopic variants found in all chromosomes, with sizes that varied from 10 Kbp to 24 Gbp (SD = 870, mean = 228) and contained zero to 181 genes (SD = 9, mean = 3), of which zero to 96 (SD = 4 mean = 1) are genes cited in the OMIM database (OMIM genes) (see supplementary information 3).

### Long contiguous stretches of homozygosity in the samples

In total, 953 CMA results whose files were available and accessible for the LCSHs study were analyzed. The majority (91%) of CMAs had at least one autosomal LCSH (≥ 3 Mbp), resulting in a total of 3445 LCSH identified in 865 individuals. Only 88 CMAs did not show any LCSH (≥ 3 Mbp). Of the total, 59% (565/953) had only LCSH below 5 Mbp, while 31% (300/953) had one or more LCSH ≥ 5 Mbp.

### LCSH leading to suspected UPD

**Figure 4:**
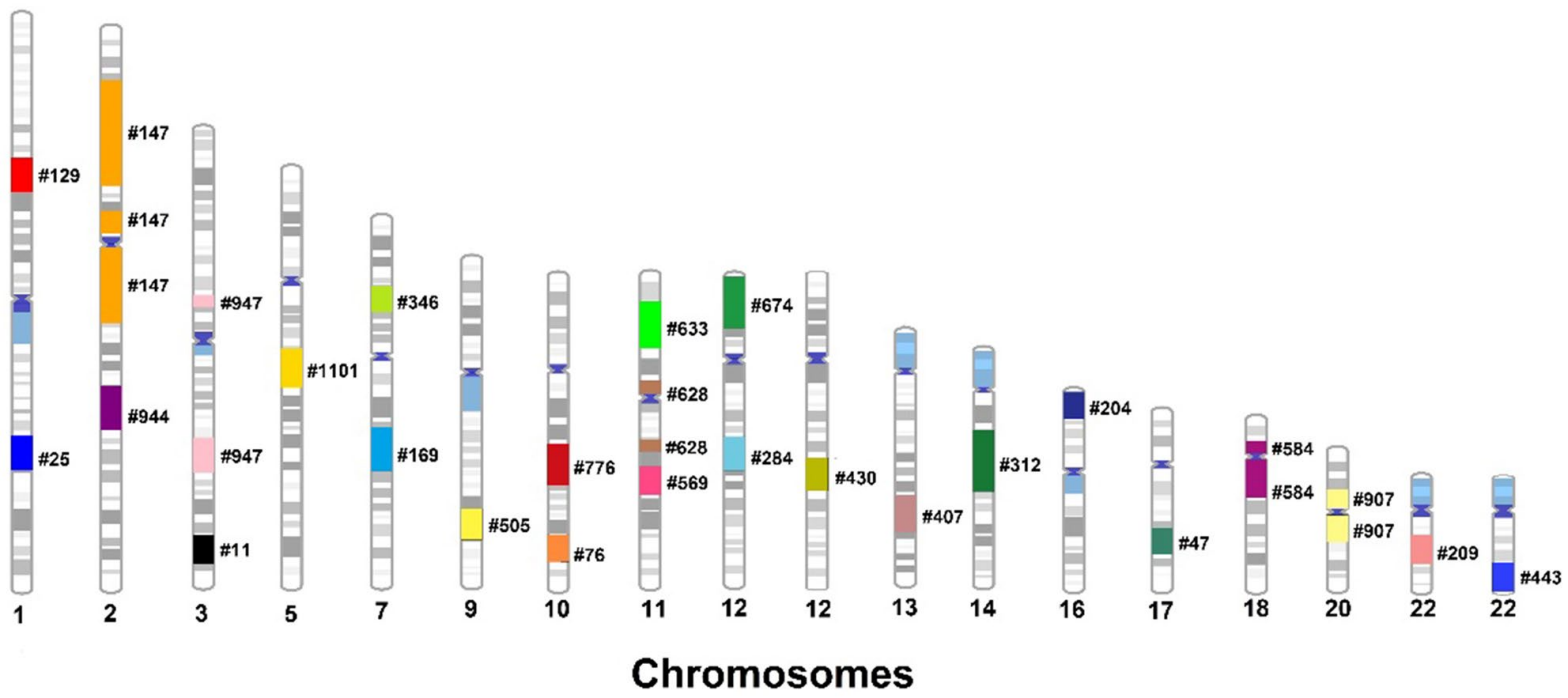
Chromosomal distribution of the 27 cases with LCSH (single or sum) ≥ 10 Mbp restricted to one chromosome, suggesting putative UPDs.

In 27 individuals (~ 2.8%) of the 953 CMA analyzed, which include 11 previously published cases^28^ the LCSH suggested a potential UPD (Table 5 and Fig. 4).

**Table 5:** Cases with potential UPDs, where a single autosomal chromosome presented LCSH(s) over 3 Mbp, that that alone or in addition of LCSHs ≥ 3 Mbp reached a size of ≥ 10 Mbp with no other LSCH over 5 Mbp on any other autosomal chromosome.

Case	Chr	UPD segment (isodisomy)	Size Mbp	Other findings	Phenotype
#25*	1	1q25.3q31.3 (182,537,598–197,949,082)	15.4	Parental origin unknown 1 PCNV on chrX*	Male, 16 yrs., DD, ID, SLD, FD, obesity
#129*	1	1p31.3p31.1 (61,620,929–76,755,163)	15.1	Parental origin unknown Without rare CNVs	Male, 4 yrs., DD, SLD, ASD
#147*	2	2p12p11.2 (9.9 Mbp; 79,211,952–89,129,064) & 2q11.1q14.3 (33 Mbp; 95,341,387–128,342,675) & 2p24.1p14 (45.9 Mbp; 22,170,065–68,067,589)	88.8	Parental origin unknown Without rare CNVs	Male, 4 yrs., DD, ASD
#944	2	2q24.1q31.1(155,368,924–174,708,199)	19.3	Parental origin unknown 1 PCNV on chr 7 Table 2	Suspected Williams-Beuren Syndrome
#11	3	3q26.32q28(176,695,771–189,044,675)	12.3	Parental origin unknown Without rare CNVs	Male, 6 yrs., bilateral cleft lip/palate, iris coloboma, blepharophimosis, camptodactyly, patent ductus arteriosus in the past, spina bifida, cerebral ventricle asymmetry
#947	3	3p13p12.3(5.3 Mbp; 72,016,624–77,325,155) & 3q22.2q25.1(15.4 Mbp; 133,992,740–149,438,082)	20.8	Parental origin unknown Without rare CNVs	Fem, 8 mo., IUGR, DD, FD macrocephaly, short stature, small hands/feet, hypoplastic external genitalia
#1101	5	5q14.1q15(77,967,561–94,997,034)	17	Parental origin unknown Without rare CNVs	Male, 6 years, ASD
#169*	7	7q21.13q31.1(90,678,991–109,653,423)	19	Parental origin unknown 1 PCNV on chr 18*	Fem, 9 yrs., FD, learning difficulties, short stature, ophthalmopathies
#346*	7	7p14.3p14.1 (29,374,797–40,699,189)	10.6	Parental origin unknown Without rare CNVs	Male, 15 yrs., DD, severe ID, epilepsy, short stature, absent speech, gastroesophageal reflux and cerebellar atrophy
#833	8	8q13.3q22.1(70,942,228–94,406,882)	23.4	Parental origin unknown 1 LPCNVs on chr 1 Table 3	Male, 2 yrs. 8 mo., ASD
#505	9	9q31.2q33.1(108,394,893–122,047,673)	13.6	Parental origin unknown Without rare CNVs	Female, 12 years, DD, ID, SLD
#76*	10	10q25.2q26.13 (112,544,654–124,513,498)	12	1 PCNV on chr 7*	Male, 12 yrs., DD, mild ID, ASD, FD
#776	10	10q22.1q23.31(72,616,063–91,065,521)	18.5	Parental origin unknown Without rare CNVs	Fem, 5 yrs., ASD. Likewise affected sister and brother with ASD, with unremarkable microarray results
#569	11	11q14.1q21(83,339,664–95,895,139)	12.6	Parental origin unknown Without rare CNVs	Fem, DI, DF, microcephaly, atopic dermatites
#633	11	11p15.3p13(11,473,107–32,068,176)	20.6	Parental origin unknown 1 LPCNVs on chr 6 (Table 3)	Male 5 yrs., ASD
#628	11	11p11.2p11.12(5.7 Mbp; 45,853,773–51,550,787) & 11q13.4q13.5(5.2 Mbp; 71,543,708–76,752,248)	10.9	Parental origin unknown Without rare CNVs	Fem, 9 yrs., ASD
#674	12	12p13.33p12.1(257,936–22,766,988)	22.5	Parental origin unknown Without rare CNVs	Male, 8 yrs., macroglossia, protruding tongue, laryngeal alterations, closure of the posterior pharynx, laryngotracheomalacia, possible Di George syndrome, peripheral pulmonary stenosis
#284	12	12q15q21.31(69,859,080–84,755,083)	14.9	Parental origin unknown Without rare CNVs Normal karyotype	Male, 10 yrs., DD, ID, FD, obesity, SLD, hypotonia, high palate, clinodactyly, long 2nd and 3rd toes, foot polydactyly, unilateral cryptorchidism, retinitis pigmentosa
#430	12	12q21.2q21.33(78,736,693–92,566,637)	13.8	Parental origin unknown Without rare CNVs	Fem, 4 yrs., DD, FD, short stature, protruding ears, low vision, retinal spot, intracranial calcifications
#407	13	13q22.1q31.3(75,078,803–92,192,744)	17.1	Parental origin unknown, half-sister with Down syndrome, 46,XX, add (21)(q22.3) 2 PCNVs on chr 3 and 21 (Table PCNVs)	FD, palatine cleft, upslanting palpebral fissures, Low weight, DD, abnormal growth, seizures, neuropathies, congenital cardiopathy, atrial and ventricular septal defects
#312*	14	14q13.2q23.2 (36,397,727–64,565,981)	28.1	Parental origin unknown 1 PCNV on chr. 22*	Male, 11 yrs., SDL, learning disability, FD, abnormal brain structure
#204*	16	16p13.3p13.13 (12.5 Mbp; 89,560–12,548,052)	12.5	Parental origin unknown Without rare CNVs Normal Karyotype	Fem, 2 yrs., IUGR, oligohydramnios, low birth weight, low stature, hypotonia, camptodactyly, DD, SLD, trigonocephaly, epicanthus, downslanting palpebral fissures, atrial septal defect
#47*	17	17q22q24.2 (53,332,043–65,633,600)	12.3	Parental origin unknown Normal karyotype 1 mosaic PCNV on chr X, (contribution)	Fem, 8 yrs., FD, abnormal eyelashes, widow's peak, supernumerary nipple, short stature, anomalies of upper and lower limbs
#584	18	18p11.22p11.21(5.2 Mbp; 9,990,161–15,143,714) & 18q11.1q12.2(17.5 Mbp; 18,540,834–36,061,962)	22.7	Parental origin unknown 1 VUS chr 4	Male, 1 year and 10 months, DD and macrocephaly
#907	20	20q11.21q13.11(12.5 Mbp; 29,510,307–42,027,093) & 20p12.1p11.1(8.8 Mbp; 17,489,413–26,266,313)	21.3	Parental origin unknown Without rare CNVs	Male, DD, deafness, ocular anomalies and oral cleft
#209	22	22q12.1q13.1 (26,504,838–40,021,614)	13.5	Parental origin unknown Without rare CNVs	Male, 5 yrs. 8mo., DD, SLD, ID
#443*	22	22q13.1q13.33 (37,977,281–51,157,531)	13.2	Parental origin unknown 1 PCNV of 2.8 Mbp (× 4), partially overlapping with this probable UPD.*	Male, 2 yrs., low weight, short stature, FD, DD, mongolian spots, poor ear development, SLD, ASD, disturbed behavior, agressive

Identified in previous work *(Chaves et al., 2019)^28^.

### Consanguinity

Analysis of LCSH distributed across multiple chromosomes indicated some degree of inbreeding in 36.5% (348/953) of cases, with over 24% suggesting seventh- to sixth-degree parentage (as third cousins); 7.2%, fifth grade (eg, second cousins); 1.8%, fourth grade (distant first cousins); 1.8%, third degree (first cousin; half-uncle with niece); 0.6%, second-degree (half-siblings, uncle-niece, double cousins) and in two cases (0.2%) parental kinship suggested incest as it is a coefficient of first-degree inbreeding [father (mother) /daughter (son), full siblings].

Clinically more relevant first-to-fifth-degree kinship was suggested by ~ 11.5% of cases.

### LCSH with frequency ≥ 5%

Due to the scarcity of information about common LCSH in the Brazilian population in previous work we decided to explore the data from this affected cohort to identify frequent LCSH in the population of Santa Catarina, which we consider to potentially be non-causal for the developmental issues of the patients^28^, and now we revise the findings with a larger sample.

The frequency of 5% or more to consider a recurrent LCSH as a common finding in the population of southern Brazil was decided on an empirical basis. This threshold was established to ensure a significant safety margin compared to the 1% threshold used for considering a Single Nucleotide Polymorphism (SNP) as a common variant in the population. This choice was made because analyzing an affected population can introduce bias. However, it is still possible that certain autozygous haplotypes act in conjunction with other genetic variations to manifest the phenotype.

**Figure 5:**
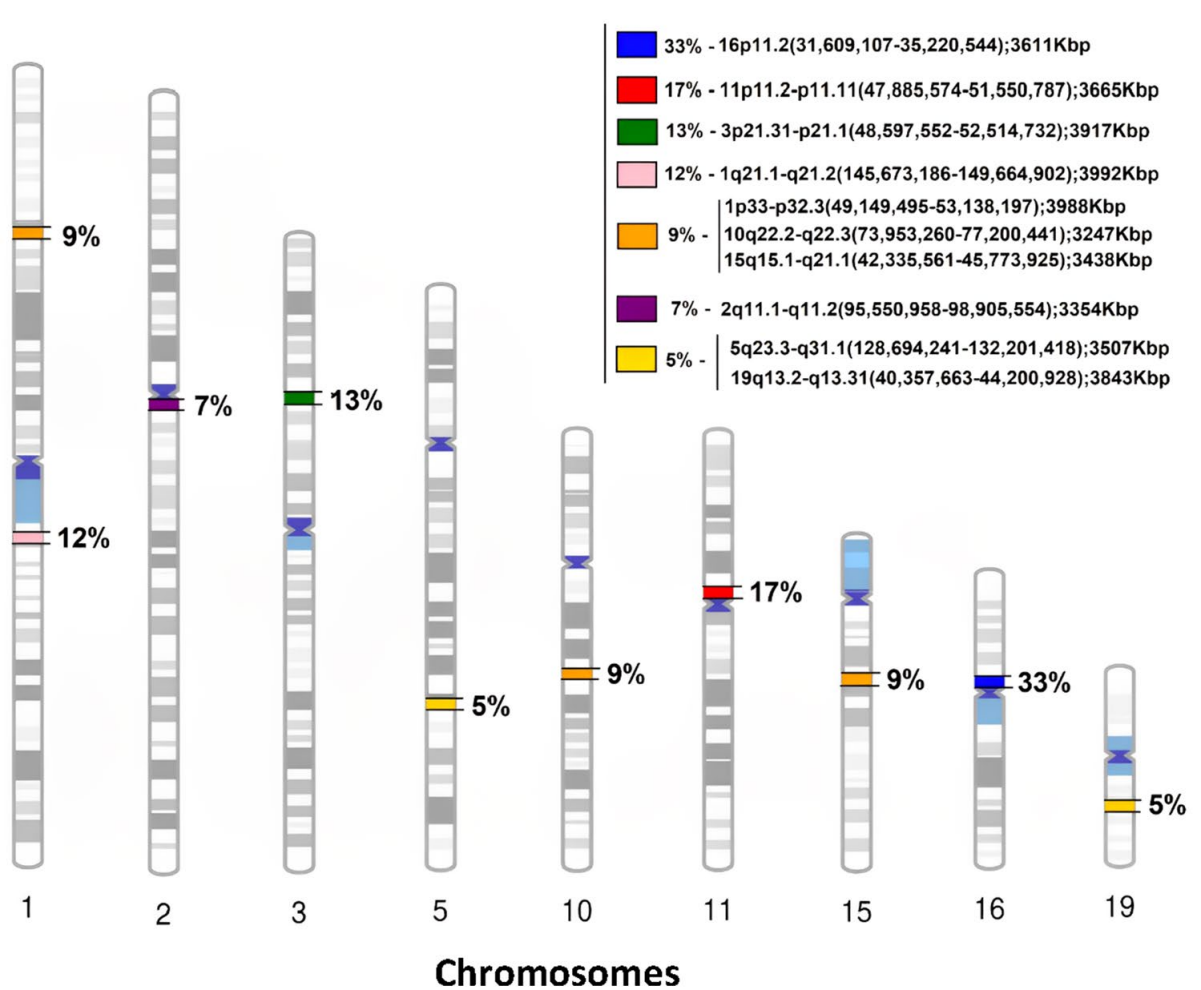
Visualization of the chromosomal locations of the LCSHs in autosomal chromosomes considered common (frequency ≥ 5%) identified among 917 CMA results.

The LCSH identified as frequent, potentially representing regions of low recombination that can maintain ancestral haplotypes identical by descent, are shown in Table 7 and Fig. 5.

## Discussion

This expanded retrospective cohort study involved 1012 patients with neurodevelopmental disorders (NDDs) and congenital anomalies (CAs) from the state of Santa Catarina. A total of 206 pathogenic copy number variations (CNVs) were identified in 170 individuals, resulting in a diagnostic yield of 17%. This diagnostic yield is almost the same as the 18% obtained in our first study^33^ and within the range of 15% to 20% of the diagnostic rate reported in the literature for patients with NDDs^33,40–52^.

It is important to highlight that out of the 173 cases with pathogenic CNVs, 32 cases had a previous abnormal karyotype result, which prompted the CMAs to identify the DNA sequences involved. Excluding the 32 cases with known abnormal karyotypes, the diagnostic rate drops to 14%. The chromosomal microarray (CMA) was essential in discovering altered sequences in abnormal karyotype results, offering unexpected insights into discrepancies compared to what a karyotype suggests. The CMA allows for scrutiny, and sometimes it reveals deletions in chromosomes where the karyotype suggests additions or additions when the karyotype suggested deletions.

In our previous work, which includes part of the current cohort, we extensively discussed the usefulness of classical karyotyping as a complement to CMA results (and vice-versa), exemplified by 17 cases with altered chromosomal results and their respective PCNV findings, including the case #687 illustrated above^33^. We can only underscore the importance of having both classical karyotype results and CMA results. They provide valuable clues about the processes leading to pathogenic changes and are crucial for genetic counselling^53,54^. Unfortunately, as CMA testing becomes more prevalent, classical karyotyping is performed less frequently, everywhere. They should at least be conducted for the child and parents when results indicate a pathogenic CNV or a potential UPD. Achieving this goal is desirable, but unattainable in most (not privileged) settings. Few cases will have access to both investigations, and even fewer will have the opportunity to investigate parents and other family members.

### CNVs

Our analysis revealed pathogenic CNVs across all human chromosomes, with more than one causative variant identified in 15% of individuals. Deletions accounted for the majority (64%) of all detected pathogenic variants, consistent with the findings of others^55^, whereas for VUS the deletions represented only 34%.

Our findings indicate a higher incidence of pathogenic variants on chromosomes 1, 3, 19, and X, with 17, 16, 15, and 18 PCNVs, respectively. This contrasts with the results of previous studies^23–26^ (see Supplementary information 1- Pathogenic CNVs per chromosome).

The sizes of the PCNVs, the number of genes they covered, and the number of OMIM genes associated with these CNVs to those of the VUS and non-causative (benign) CNVs, show a statistically significant difference with *P* < 0.0001 (according to Tukey's Multiple test) (Fig. 3A1 and Supplementary information 2). This is comprehensible, since larger CNVs, with more genes, in particular with more genes related to disease or known to drive important cellular processes will have a higher impact, which tends to be greater for absence of gene copies than for their excess.

As depicted in the circus ideogram (Fig. 2), pathogenic CNVs tend to be situated near telomeres in most chromosomes. This is expected since subtelomeric regions are prone to rearrangements, given that only one chromosomal breakpoint is required to initiate a submicroscopic abnormality^56^.

Pathogenic CNVs are also known as recurrent and non-recurrent. While non-recurrent pathogenic CNVs occur sporadically in the genome, with probable origins in replication errors or DNA repair mechanisms, they cover different gene contents and consequently present variable phenotypes^55–57^. Recurrent pathogenic CNVs, in turn, are associated with known and characterized microdeletion and microduplication syndromes. Recurrence of these CNVs is mediated by non-allelic homologous recombination between locus-specific low copy repeats (LCRs)^58,59^.

We have identified a total of 71 individuals with known syndromes that are associated with 72% of pathogenic CNVs. Among them, the most common were Angelman/Prader Willi syndrome, Di George syndrome (0.7%), 1p36 deletion syndrome (0.6%), 16p11.2 deletion syndrome, and Cri Du Chat syndrome (0.5%) (Supplementary Table 2).

### Phenotypic characterization

Characterizing phenotypes is a crucial step in investigating the genetic etiologies of developmental disorders, helping to identify the role of the genes involved, as Moeschler and Shevell's (2014)^60^ emphasized in their systematic review about the investigation of children with global developmental delay and intellectual disability.

In our cohort, the phenotypic characterization revealed a predominance of phenotypes related to NDs, accounting for 85% of cases, similar to findings reported by others^55,59,61^, with 83% of the individuals presenting ID and/or DD. In 56% of cases DD was present, while ID was mentioned for 33%. Autism Spectrum Disorders were present in 33% of the cohort, in 14% of the cohort we had “isolated” ASD (without ID and without DF). It's worth noting that 42% of the cohort was under 5 years of age, which is below the typical age range for diagnosing ID and eventual deficits are diagnosed as DD. Nevertheless, even considering that many individuals with DD are not necessarily intellectually deficient, it is still possible to estimate the prevalence of Intellectual Disability (ID) by including individuals with both DD and ID, because it is known that most individuals with DD in early childhood will later receive a diagnosis of ID^62^.

Along with major neurodevelopmental phenotypes, many individuals exhibit syndromic features (56%), such as congenital anomalies or malformations, and most (47% of all) had atypical facial appearance (facial dysmorphism). Other comorbidities, such as psychiatric or behavioural problems, and variations in physical parameters, like height or body weight, were less frequently reported.

With a larger sample than in our previous study, the univariate analysis confirmed our first findings, showing a significant association for the presence of pathogenic CNVs with autism spectrum disorders (in this case, with a lower presence), facial malformations/dysmorphisms and genitourinary anomalies/malformations. Obesity and short stature, that were significantly related as second relevant phenotypes when the cohort was smaller^33^, lost their significance in the now larger sample. Now developmental delay, intellectual disability, limb anomalies, low weight, heart anomalies/malformations and motor development delay gained in significance (see Supplementary Information 3).

However, even with such an extended sample, there is not one phenotype or group of neurodevelopmental or malformation phenotypes with sufficiently robust evidence as to justify a preferential CMA testing decision. Additionally, we are aware of our limitations in obtaining standardized phenotype data. This is mainly because there is no standardized phenotype collection and annotation among medical doctors, most of whom are not geneticists and have limited access to genetic tests for follow-up genome sequencing or mutation investigation.

In the State of Santa Catarina, which has approximately the size of Hungary and close to 7.6 million inhabitants, there are only a few (about five) medical geneticists, most of whom practice in Florianópolis, the state capital. Consequently, many patients come from distant areas or are referred for testing by medical doctors outside the main city, without the opportunity to consult with a medical geneticist. A comprehensive and standardized reassessment in all cases, which is currently beyond our capabilities, would be crucial for confidently confirming the phenotype findings and, not to mention, aiding in the interpretation of the CNVs found.

### ASD cases

For the 333 cases of cohort who were diagnosed within the ASD, the ages ranged from a few months to 34 years, with a male predominance of 3.7:1. This is interesting, because when considering the male to female ratio of the whole cohort, the proportion is 1.55:1 and when the cases that mention ASD phenotypes in the clinical description are excluded, the male to female ratio is 1.1:1. We are aware that the cases did not undergo a standardized clinical assessment for ASD. However, the ratio of about 4 M:F is well established in the literature, and has led to specific reviews on sex differences in ASD^63–68^.

Based on the clinical data which we could obtain, 29% of the individuals (79 aged 5 or more; 17 under 5 years of age) of our ASD cohort also had dysmorphic features (DF), a term that we used to include facial dysmorphia and/or congenital anomalies. When DF were present, we considered them to be syndromic ASD cases, that could have ID or not.

Like the diagnosis of ASD, the diagnosis of ID did not follow a standardized protocol. Some individuals underwent detailed cognitive tests, and others were diagnosed by doctors based of several criteria, this can be seen on Tables 1 and 2, where in most cases only ID is mentioned, without the degree of the ID (mild, moderate, severe). Within the 256 individuals with ASD aged 5 or more, 68 (27%) had some degree of ID. Isolated ASD, which we use to define the non-syndromic patients without ID, comprised 44% (145/333) of the cohort.

According to Rosti et al. (2014)^69^, approximately 75% of ASD were essential (non-syndromic) cases, whereas 25% are syndromic. Lovrečić et al. (2018)^70^, reported a proportion of 41% of isolated ASD, 41% with DD and 19% with complex (syndromic) phenotypes when studying a cohort of 150 ASD cases.

There are wide differences within the published prevalence of ID among autistic individuals, Chiurazzi et al. (2020)^71^ mentions a coexistence of 70% of cases with ASD with ID, while 40% of cases with ID have ASD^72^. The Autism and Developmental Disabilities Monitoring Network (ADDM) funded by the CDC, states that about one third of individuals (35.2%) of the ASD spectrum also have some degree of ID (CDC—Autism Spectrum Disorder, last reviewed December 15, 2022).

There are sex differences among the subclasses of ASD. Whereas the male:female ratio for the whole ASD cohort is 3.8:1, for syndromic ASD it is 2.9:1. In syndromic ASD with ID it is 4.1:1; syndromic ASD w/o ID, 2.3:1. For non-syndromic with ID it is 3:1, and for isolated Autism (non-syndromic w/o ID) it is 4.8:1.

CNVs were found in 90% of the 333 CMAs analysed, and 38 CNVs interpreted as pathogenic were detected in 35 cases with ASD, resulting in a diagnostic yield of 10%, lower than the diagnostic rate for the whole cohort (17%), but within the range of 8 to 22% cited in the literature for other ASD cohorts^16,70,73–85^. And without the ASD cases, the diagnostic rate of the cohort increases to 20%.

Within the 35 cases with pathogenic CNVs, 4 were among the 9 patients that had previous abnormal karyotype results, for which the CMA test was requested to identify the DNA sequences involved. Excluding the 4 cases with known abnormal karyotypes, the diagnostic rate drops to 9%, however, the diagnostic yield was considered 10% because the CMA was essential to discover the altered sequences in the abnormal karyotype results.

### Recurrent and rare CNVs in ASD

The pathogenic CNVs found in this study and the reported phenotypes of the respective patients are detailed in Table 3. We highlight the genetic syndromes involved with these alterations, which were identified in our cohort, in addition to the most common syndromes in ASD, which involve the chromosomal regions 15q11-q13, 16p11.2 and 22q11.2^86–92^, such as the 15q13.3 Microdeletion Syndrome (#612001), Chromosome 16p.11.2 Deletion Syndrome (OMIM# 611913 ; n = 2), Distal 16p11.2 Deletion Syndrome (#613444) (in 2 cases), Distal 22q11.2 Microduplication Syndrome (# 608363) and Angelman/Prader-Willi Syndrome (*600162).

Also rarer syndromes like 1q21.1 Microduplication Syndrome (#612475), 2q37.3 Microdeletion Syndrome (#600430), Williams-Beuren Region Duplication Syndrome (#609757, n = 2), 9p Deletion Syndrome (#158170), Distal 13q Deletion Syndrome (#613,884), Temple Syndrome (#616222), Partial Trisomy 16p13.3 Syndrome, Potocki-Lupski Syndrome (#610883), Distal Chromosome 18q Deletion Syndrome (#601808), 18q Deletion Syndrome (#601808), Schinzel Giedion Syndrome (#269150), 21q22.12 Microdeletion Syndrome, 22q13 microdeletion/Phelan-McDermid syndrome (OMIM# 606232; n = 2), MECP2 Duplication Syndrome (#300260), Triple X Syndrome and XYY Region Syndrome have been associated to ASDs.

Among the pathogenic CNVs detected in our study, the ones with the highest frequency in the literature, based on data from the SFARI bank, are the 16p11.2 microdeletion (108 entries), followed by the duplication of 7q11.23 (85 entries), the 16p13 microduplication. 3p12.3 (73 entries), the Xq28 microduplication (59 entries), the 15q11.2q13.1 microdeletion (56 entries), the 22q13.33 microduplication (54 entries), and the 17p11.2 microduplication (45 entries). And identical to the findings of Li et al. (2015)^93^, in our study chromosomes 15, 16 and 22 together contributed to more than 25% of pathogenic CNVs.

Among the rarer findings, based on the SFARI database we have: Case #66, carrying a 22 Mbp microduplication at 15q25.1q26.3(80,304,866–102,429,040), with no SFARI entry for the locus; Case #345, a 2.7 Mbp microdeletion at 14q32.2q32.31(100,095,248–102,755,064), with two entries; the case #385, with a 4 Mbp microdeletion at 21q22.12q22.2(35,834,713–39,831,660), with only one entry; Case #443, carrying a heterozygous microduplication (4×) of 2.8 Mbp at 22q12.3q13.1(35,888,588–38,692,765), with two entries for duplication and 4 for locus deletion; Case #455, which is a 26 Mbp duplication in Yp11.31p11.2-Yq11.23(2,650,140–28,799,937), with 6 entries from a single study^91^; In case #751, with an 8.3 Mpb microdeletion at 18q12.2q21.1(36,210,635–44,530,609), with a single entry; Case #873, a 10 Mpb microdeletion at 13q33.2q34(105,020,842–115,107,733), with 11 entries. And case #1107, with altered karyotype, as previously mentioned, presented a deletion of 15 Mbp in 9p24.3p22.3(208,454–15,424,987), with two entries, one deletion and one duplication.

When it comes to submicroscopic chromosomal alterations, both deletion and duplication of CNVs can result in decreased gene expression by gene disruption, whether gene duplications can also lead to overexpression of genes.

As discussed by Velinov^94^, the detection and interpretation of recurrent CNVs, which are often associated with ASD, facilitates post-test genetic counseling, since one can safely conclude the genetic etiology by associating the CNVs with the clinical characteristics of the patient. In most cases, particularly when the parents are unaffected, it is more likely that pathogenic CNVs have their "de novo" origins. This occurs due to events such as errors during meiotic recombination, early illegitimate mitotic recombinations, or due to repairs to DNA double-stranded breaks during the first divisions of embryonic cells^95^.

On the other hand, pathogenic CNVs can also originate from the consequences of a balanced chromosomal translocation in the genome of the parents, according to Nowakowska et al. (2016)^96^, it is advisable to test the parents of individuals with large pathogenic CNVs, through the classic karyotype, since that balanced translocations cannot be identified by CMA and carry a high risk of recurrence.

### Influence of dysmorphic features and/or ID in the diagnostic rate

Although the diagnostic rate for several phenotypic groups was higher than the 10% of diagnostic rate found in the ASD cohort, only the diagnostic yield of 16% for syndromic ASD was confirmed as significant by univariate analysis (*p* ≤ 0.05, OR = 2.43) (Fig. 3C).

Several studies have investigated the diagnostic yield of CMAs and genome sequencing techniques in cohorts with neurodevelopmental disorders and, even though with a large diagnostic variation when whole genome or exome sequencing is applied, syndromic patients tend to have significatively higher probability for a positive diagnostic result^33,97,98^. Specifically for ASD, the mean diagnostic yield is usually lower than for a typical neurodevelopmental cohort. However, among autism subtypes, higher diagnostic usually occurs when ASD is syndromic accompanied with other features and is syndromic (or complex) ASD^78,99^.

### LCSHs

In 2006, Li et al. (2006)^35^, indicated that LCSH were more common in the human genome than was considered at the time and that they could have an impact on many fields of genetic studies. We now know that LCSH are one of the most common types of genomic traits in humans, being observed throughout the human genome as a consequence of inbreeding or evolutionary forces^22,26,100–102^.

Previously we described the analysis LCSHs in 430 cases that are part of this cohort^28^. Now, considering the whole cohort, we found that 91% of the individuals have at least one autosomal LCSH ≥ 3 Mbp as revealed by their CMAs tests.

Potential UPDs were found in 2.8% of the CMAs of the cohort, similar to the 2.6% we found in or previous work^28^. The frequency of potential or confirmed UPDs found among published cohorts varies largely among studies. Investigating 214,915 trios, from the 23andMe sequencing dataset, representing a non-clinical general population, the authors found 105 cases of UPD estimating that UPD occurs with an overall prevalence rate of roughly 1 in 2000 births or 0.05%^103^. The frequency of UPDs found in studies that used exome sequencing of patient-parent trios of large clinical populations for all sorts of genetic conditions is higher and oscillates between 0.2 and 0.6%^104–106^. The investigation for UPDs with whole genome sequencing of 164 parent–child trios in a more selected cohort, an Irish cohort with rare disorders, found 3 UPDs a frequency of 1.8%^105^.

Using CMA platforms with distinct SNP density and in clinical populations with distinct ethnic backgrounds, the reported potential UPD rate oscillates from 1 to over 4%^23,106–109^.

We want to emphasize once again that CMA technology can only detect UPD regions in cases of isodisomy; it cannot identify UPDs with total heterodisomy. In a complete UPD, whether it's isodisomic, iso/heterodisomic, or entirely heterodisomic, both homologous chromosomes will exhibit the gende-specific imprinting of the sole transmitting parent across their entire length. It's also important to remember that long, uninterrupted stretches of homozygosity may also result from homologous repair through a breakage-induced DNA replication mechanism, which, in contrast, can originate segmental UPDs^110^.

When considering the processes that lead to UPD, it's worth noting that among the 27 cases with LCSH suggesting a potential UPD, eight also had PCNVs that were either considered responsible or partially responsible for their clinical conditions. Additionally, three presented VUS, including two with LPCNVs.

One exception is case #584, which had a PCNV spanning 2.8 Mbp (4×) and overlapped with approximately 1 Mbp of the homozygous region associated with the putative UPD, whose complex origin hints to a real segmental UPD. All other CNVs were located on chromosomes unrelated to the identified UPD. We did not detect any traces of mosaicism involving the affected chromosome in any of the cases, which could have suggested a trisomy rescue.

When a potential UPD is found on one of the chromosomes related to imprinting disorders, like chromosomes 6, 7, 11, 14, 15 or 20, and the phenotype of the patient fits the potential imprinting disorder phenotype, the follow-up is straightforward^111,112^. However, most often the UPDs are on chromosomes without imprinted regions and sequencing of the isodisomic region should be considered because it often unmasks a homozygous deleterious variant inherited from a heterozygous parent^107^.

Out of the 27 potential UPD cases identified in our study (Table 5 and Fig. 4), only seven were associated with chromosomes known for imprinting disorders^110^. Cases #169 and #346 on chromosome 7, as well as case #312 on chromosome 14, have been previously discussed^28^. Among the cases with potential UPD-like LCSH patterns on chromosome 11, case #633 has a PCNV identified as the causal factor for its clinical condition, and cases #569 and #628 do not exhibit the hallmark phenotypes typically associated with Beckwith–Wiedemann overgrowth syndrome caused by UPD(11)pat or Silver-Russel Syndrome caused by UPD(11)mat. The same is true for case #907 on chromosome 20, whose available phenotypes do not correlate at all with the imprinting disorders of these chromosome.

### Consanguinity

Approximately 24% of the CMAs revealed an LCSH pattern suggesting a distant familial connection (sixth or seventh degree) among the parents of patients affected by NDs. As we've previously mentioned, these findings may be indicative of regional immigration patterns and intermarriage among immigrants in southern Brazil. When the relationship suggested by the LCSH is distant and more associated with the endogamous characteristics of the population, the likelihood of clinical significance decreases.

More significant is the fact that in 11.5% of the CMAs, the LCSHs indicated a first to fifth-degree parental relationship between the parents. These cases are more likely to have a clinical impact because the closer the parentage, the higher the proportion of shared alleles, increasing the risk of inheriting two copies of an autosomal recessive (AR) mutation^24^. We provide an in-depth discussion of the impacts and relevance of these findings in a previous publication^28^.

As shown in Table 6, two patients exhibit homozygosity, indicating potential first-degree relatedness among their parents. These results are communicated to the referring physicians by the diagnostic laboratory. It is the responsibility of these physicians to follow the appropriate protocols for these cases.

**Table 6:** Details the results referring to the 4.3% of cases that suggested kinship from first to fourth grade.

Cases	∑ of LCSH (Mbp)	Possible parental relationship	Degree of kinship	Coefficient of inbreeding (F)	LCSH (IBD) expected not tested (∼%)
#194	760	Father (mother)/daughter (son); complete siblings	First	0.264	25
#834	1.053			0.37	25
#271	334	Half-brothers; uncle (aunt)/niece (nephew); double first cousins; grandfather/granddaughter	Second	0.116	12.5
#1068	403			0.14	12.5
#918	285			0.10	12.5
#297	314			0.109	12.5
#380	346			0.121	12.5
#220	402			0.139	12.5
#187	196	First cousins	Third	0.068	6
#275	225			0.078	6
#395	136			0.047	6
#412	123			0.043	6
#413	162			0.056	6
#419	181			0.063	6
#354	193			0.067	6
#364	165			0.057	6
#540	196			0.068	6
#645	238			0.082	6
#730	137			0.047	6
#754	204			0.070	6
#766	136			0.04	6
#823	183			0.063	6
#910	248			0.086	6
#1088	227			0.079	6
#1103	239			0.082	6
#157	62	First cousins once removed	Fourth	0.022	3
#273	110			0.038	3
#287	96			0.033	3
#311	82			0.028	3
#378	93			0.032	3
#412	123			0.042	3
#506	68			0.023	3
#546	73			0.025	3
#612	88			0.030	3
#614	81			0.028	3
#663	90			0.031	3
#676	106			0.036	3
#770	75			0.026	3
#789	123			0.042	3
#806	74			0.025	3
#905	66			0.023	3
#1011	79			0.027	3

LCSH with frequency ≥  5%.

For one patient (#1068) where a second-degree relatedness is suggested among his parents (Table 6) a PCNV was identified in chr 15 (Table 2). This patient presents a complex syndromic phenotype that extends beyond the typical manifestations usually associated with this deletion, which are mainly related to ASD, DD and behavioural issues, suggesting the participation of a causal autosomal recessive development gene.

### LCSH considered common (frequency ≥ 5%)

As extensively discussed in Chaves et al. (2019)^28^, identifying and knowing the most common (recurrent) LCSH allows us to focus the analysis on the most clinically significant LCSH. Following the same reasoning and criteria of our initial study, in this new analysis, we have identified ten LCSH ≥ 3 Mbp occurring at a frequency of 5% or higher, thus considering these LCSH as a possible common variation in our population.

All LCSH, except for 19q13.2-q13.31 (40,357,663–44,200,928), which was identified as frequent in our dataset (Table 7) have been previously recognized as common LCSH by other research groups in clinical investigations involving patients with developmental disorders^28,36–39,108^, including our previous work. These LCSH are typically considered low recombination regions, representing blocks of ancestral haplotypes, and are generally interpreted as potentially non-pathogenic.

**Table 7:** Regions of LCSH considered common (frequency ≥ 5%) identified among 917 CMA results.

Frequencies	Chr/Cytobands	Initial position	Final position	Size (Kbp)
33	16p11.2^a,b,c,d,f^	31609107	35220544	3.611
17	11p11.2-p11.11^a,b,c,d^	47885574	51550787	3.665
13	3p21.31-p21.1^a,b,c,d,e^	48597552	52514732	3.917
9	1p33-p32.3^a,c,d^	49149495	53138197	3.988
9	15q15.1-q21.1^a,d^	42335561	45773925	3.438
9	10q22.2-q22.3^a,d^	73953260	77200441	3.247
7	2q11.1-q11.2^a,c,d^	95550958	98905554	3.354
12	1q21.1-q21.2^a,c,d^	145673186	149664902	3.992
5	**19q13.2-q13.31**	**40357663**	**44200928**	**3.843**
5	5q23.3-q31.1^c^	128694241	132201418	3.507

When the beginning and/or end of the cytobands were variable, a linear position was obtained based on the median of the beginning or end. All analyses, as well as linear positions, were based on the human reference genome, version GRCh37/hg19. (a) Chaves et al. 2019, (b) Wang et al. 2015, (c) Kearney H. M. (personal communication, 2017), (d) Sanchez P. (personal communication, 2017), (e) Pajusalu et al. 2015, (f) Neta et al. 2022.

The bolded LCSH was only found in our study.

Wang et al. (2015)^37^ identified several of these regions as recurrent LCSH without clinical relevance in a cohort of patients with NDDs, including unaffected parents. Kearney HM^39^ reported them as findings occurring at a frequency > 5% in CMA readings (CytoScan HD, Affymetrix) from affected individuals. Sanchez P^38^ in an analysis of a cohort of 278 affected Hispanics reported LCSH as common when their frequency exceeded 3% in CMA samples (CytoScan HD, Affymetrix). Neta et al. (2022)^108^ reported the region we found on chromosome 16 as occurring at a frequency of 12.7% in a cohort of 100 patients with ID and/or ASD from the Midwest region of Brazil. Pajusalu et al. (2015)^36^ reported similar findings to ours on chromosomes 3 and 11 as recurrent LCSH with frequencies of 9.3% and 6%, respectively, using a minimum cutoff size of 5 Mbp, in the investigation of 2110 consecutive Estonian patients (including prenatal care and parenting samples).

In our previous research, we identified as common the regions 6p22.2p22.1 (26,340,871–30,006,805) and 20q11.21q11.23 (31,940,638–36,081,725), also reported as common by Sanchez P^38^, Kearney HM^39^, and Pajusalu et al. (2015)^36^, as well as 7q11.22q11.23 (71,997,278 -76,128,151), that had no prior report. However, they were not confirmed at a frequency ≥ 5% in this larger sample. Conversely, our previous study did not identify 5q23.3-q31.1 (128,694,241–132,201,418), also found by Kearney HM^39^, as frequent. However, in the larger cohort this LCSH now shows up at a frequency above 5%.

We found no previous report of the LCSH in 19q13.2q13.31 (40,357,663–44,200,928) that we identified now. This homozygous region is not associated with any genes known to have an imprinting pattern in humans^113^. It encompasses 148 known genes, out of which 81 are listed in OMIM, including five genes related to autosomal recessive (AR) disorders: Charcot-Marie-Tooth Disease, Type 4F (#614895), Maple Syrup Urine Disease (#248600), Neurodevelopmental disorder with hypotonia, neuropathy, and deafness (#617519), Ethylmalonic Encephalopathy (#602473), and Agammaglobulinemia 3 (#613501).

The LCSH considered frequent and common in the current study not only support the findings and discussions of our previous research but also raise the possibility that our threshold of considering LCSHs only at a frequency ≥ 5% could be too conservative. It might be a relatively safe alternative to consider a lower threshold, such as LCSHs with a frequency above 4% or 3%, as Sanchez P^38^ did.

## Conclusions

In this retrospective study, we present the largest report of microarray chromosome data (CMA) in a cohort with neurodevelopmental disorders (NDDs) and/or congenital anomalies (CAs) from Southern Brazil. We achieved a diagnosis rate of 17%, consistent with the literature (15–20%). We characterized the rare copy number variations (CNVs) that we identified and associated them with the main phenotypes presented by each patient. The interpretation of CNVs is challenging and relies on information such as frequency and characterization in affected populations, typically obtained from cohort studies with significant sample sizes.

The primary reasons for referring individuals to CMA testing in this study were developmental delay/intellectual disability and autism spectrum disorder, often accompanied by syndromic features like congenital anomalies or dysmorphic features. Certain phenotypes have been shown to predict a higher likelihood of carrying a pathogenic CNVs.

For the cases with the ASD, although our diagnostic yield of 10% for ASD is within the range reported in the literature (8–21%), it is higher (16%) when it is syndromic, associated with dysmorphic features, and lower (7%) for "isolated" ASD.

Among the 953 CMAs analysed for contiguous stretches of homozygosity (LCSH), we observed 27 large LCSH (≥ 10 Mbp, ranging from 10.6 to 88.8 Mbp) on a single autosome, suggesting a potential frequency of uniparental disomy (UPD) of 2.8%. However, the limitations of CMA underestimate the true UPD rate, as it can only suggest its presence when uniparental isodisomy is detected. The absence of methylation tests hinders confirming these findings as real UPDs and distinguishing between complete and segmental UPDs.

Regarding consanguinity, the analysis of LCSHs indicated a possible descent from first- to fifth-degree relatives in approximately 11.5% of the cohort. This information is crucial for genetic counseling, as close relatives pose an empirical risk of recurrence, potentially due to autozygous autosomal recessive (AR) mutations. In cases with affected siblings, the analysis of regions that are identical by descent (IBD) can assist in identifying the target region for investigation, particularly when employing whole-exome sequencing (WES).

We identified ten LCSHs with a frequency above 5% in individuals with NDs. Nine of these LCSH had previously been reported as common variants by other research groups, suggesting that they are likely normal population variants in Santa Catarina. It might be possible that our threshold of considering LCSHs only at a frequency ≥ 5% could be too conservative. While valuable for prioritizing clinically relevant LCSHs for analysis, a clinical contribution of this homozygous regions cannot be completely ruled out.

Overall, analysing LCSHs detected by CMA with high SNP density provides valuable information to aid in the investigation of neurodevelopmental disorders. However, these findings are mostly theoretical and suggestive, serving as guidelines for further investigations such as methylation analysis, targeted gene sequencing, or WES.

### Supplementary Information

Supplementary Information.

## Data Availability

The datasets used and/or analyzed during the current study can be requested from the corresponding author on reasonable request. However, since the patients or their caregivers signed an Informed Consent Form specifying that the data will be used only for the present study, their use for another study necessarily implies a new submission to the ethics committee of the Hospital Infantil Joana de Gusmão and depends on a new approval.

## Abbreviations

NDs: Neurodevelopmental disorders
ID: Intellectual disability
ASD: Autism spectrum disorder
CMA: Chromosomal microarrays
CNV: Copy number variants
DGV: Database of Genomic Variant
OMIM: Online Mendelian Inheritance in Man
DECIPHER: Database of Chromosomal Imbalance and Phenotype in Humans using Ensembl Resources
VOUS: Variant(s) of uncertain clinical significance
DD: Development delay
CA: Congenital anomaly
IUGR: Intrauterine growth restriction
Mbp: Mega base pairs
LCSH: Long continuous stretches of homozygosity
