# Supplementary material for: A cohort study of neurodevelopmental disorders and/or congenital anomalies using high resolution chromosomal microarrays in southern Brazil highlighting the significance of ASD
Source: Sci Rep. 2024 Feb 14;14:3762. doi: 10.1038/s41598-024-54385-2 (PMC10867078; doi:10.1038/s41598-024-54385-2)
Supplement: Supplementary file 1 — Supplementary Information. [file 41598_2024_54385_MOESM1_ESM.docx]

1. Supplementary table 1

**Table of VUS found in the cohort (INCLUDING CASES PUBLISHED IN chaves, et al, 2019).**

| Case | VUS | Microarray Nomenclature | Size (Kbp) | N° of Genes | N° of Genes in OMIM | Important Genes | Phenotype | Gender /Notes |
| --- | --- | --- | --- | --- | --- | --- | --- | --- |
| #1 | Dup | arr[hg19] 2p24.1(23,982,758-24,813,485)x3 | 831 | 18 | 7 | ATAD2B, UBXN2A | MildID, overweight | F/- |
| #5 | Dup | arr[hg19] 6q15(89,917,335-90,485,874)x3 | 568 | 7 | 4 | GABRR1, GABRR2 | MID, Mot Dif and hyperactivity | **M** |
| #6 | Dup | arr[hg19] 2q37.2q37.3(236,733,535-237,355,774)x3 | 622 | 4 | 2 | AGAP1, GBX2 | DD, convulsions and FD | **F** |
| #7 | Dup | arr[hg19] 1q44(246,324,898-246,688,599)x3 | 363 | 2 | 1 | SMYD3 | ASD, motor difficulties, convulsions and FD | M/- |
| #13 | Del | arr[hg19] 11q14.1(84,050,388-84,415,990)x1 | 365 | 1 | 1 | DLG2 | ASD, LDO, motor difficulties, FD and SLD | M/- |
| #19 | Dup | arr[hg19] 8q21.13(82,061,218-84,515,685)x4 | 2,454 | 10 | 6 | IMPA1 | DD, FD, gastroschisis bladder exstrophy, hydronephrosis and Abnormal growth | **M/** **Affected brother (#18)** |
| #21 | Del | arr[hg19] 2q13(110,504,318-111,365,996)x1 | 861 | 16 | 3 | NPHP1 | ID | **M** |
| #32 | Dup | arr[hg19] 20q13.33 | 200 | 9 | 5 | KCNQ2, CHRNA1 | Convulsions, low weight, prematurity, FD, |  |
| #40 | Del | arr[hg19] 14q24.2(73,590,938-73,776,190)x1 | 185 | 4 | 2 | PSEN1, NUMBP1 | ASD and SLD | M/- |
| #43 | Del | arr[hg19] 16q23.2(80,260,131-80,701,060)x1 | 440 | 2 | 1 | DYNLRB2, CDYL2 | MildID, ASD, motor difficulties, SLD, hyperactivity and FD | M/- |
| #50 | Del | arr[hg19] 13q12.12(60,425,635-60,688,042)x1 | 262 | 25 | 2 | SGCG, SACS | MID | **F** |
| #58 | Dup | arr[hg19] 11q22.3(102,946,063-103,827,049)x3 | 880 | 4 | 2 | DYNC2H1 | DD, LDO, Mot Dif and FD | **M** |
| #64 | Dup | arr[hg19] 9q34.3(139,381,821-140,086,032)x3 | 704 | 48 | 28 | NOTCH1 | DD, SLD, ID and FD | **M** |
| #81 | Dup | arr[hg19] 16p13.3(549,826-1,449,862)x3 | 900 | 45 | 26 | CACNA1H | SLD, convulsions and FD | **M/PCNV** |
| #82 | Dup | arr[hg19] 4q35.2(188,106,543-189,797,261)x3 | 1,691 | 5 | 1 | ZFP42 | DD and SLD | **M** |
| #86 | Del | arr[hg19] 13q21.2(60,425,635-60,688,042)x1 | 262 | 2 | 1 | DIAPH3 | DD and LDO | F/- |
| #89 | Dup | arr[hg19] 9p24.3(319,876-517,446)x3 | 198 | 2 | 2 | DOCK8 , KANK1 | ASD, SLD, motor difficulties and FD | F/- |
| #109 | Dup | arr[hg19] 4q31.1(139,758,054-139,988,340)x3 | 230 | 2 | 1 | CCRN4L | DD and FD | **M** |
| #112 | Dup | arr[hg19] 9p13.3(34,211,157-34,395,294)x3 | 184 | 5 | 3 | UBAP1, NUDT2 | SID, Aut, convulsions, SLD, mot dif and FD | **M** |
| #117 | Dup | arr[hg19] 19q13.33(48,206,212-48,431,081)x3 | 224,869 | 25 | 7 | CORD2 |  | **F/-** |
| #136 | Dup | arr[hg19] 4q28.1q28.2(128,789,028-128,891,808)x3 | 102,78 | 3 | 2 | PLK4 | Low weight, short stature, IUGR, FD, thin hair | **F/-** |
| #138 | Dup | arr[hg19] 6p21.2(37,609,169-37,868,513)x3 | 259,344 | 2 | 2 | MDGA1 | Prematurity, DD, polydactyly, aggression, | **M/-** |
| #144 | Del | arr[hg19] 8q13.1q13.2(67,999,679-68,190,627)x1 | 190,948 | 2 | 2 | CSPP1 | DD, SLD, ID and FD | **F/-** |
| #178 | Dup | arr[hg19] 11q23.3(117,000,284-117,312,611)x3 | 312,327 | 10 | 7 | DSCAML1, CEP164, BACE1 | ASD, FD, macrocephaly | M/- |
| #180 | Del | arr[hg19] 16p13.3(6,243,228-6,835,898)x1 | 592,67 | 1 | 1 | RBFOX1 | DD, hypothyroidism | **M/-** |
| #215 | Del | arr[hg19] 3q26.33(179,508,262-179,621,954)x1 | 113,692 | 1 | 1 | PEX5L | Motor delay, DD, ID, ASD and ADHD | M/- |
| #223 | Dup | arr[hg19] 15q24.1(72,838,805-73,581,757)x3 | 742,952 | 8 | 4 | BBS4 | Short stature, IUGR, DD, MID and FD | **M/*3Pv** |
| #223 | Dup | arr[hg19] 3p26.3(255,645-1,510,822)x3 | 1,255,177 | 2 | 2 | CTN6, CHL1 | Short stature, IUGR, DD, MID and FD | **M/*3Pv** |
| #223 | Dup | arr[hg19] 6q25.3(156,488,875-158,534,725)x3 | 2045,85 | 9 | 4 | SNX9, ARID1B | Short stature, IUGR, DD, MID and FD | **M/*3Pv** |
| #245 | Dup | arr[hg19] 14q12(26,490,666-27,520,832)x3 | 1,030,166 | 2 | 1 | NOVA1 | Obesity, encephalopathy, CAs, DD and FD | **F/-** |
| #248 | Del | arr[hg19] 10q23.1(87,392,282-87,791,684)x1 | 399,402 | 1 | 1 | GRID1 | Abnormal brain structure, DD, | **M/-** |
| #255 | Del | arr[hg19] 10q23.1(87,691,467-87,843,627)x1 | 152,16 | 1 | 1 | GRID1 | DD | **M/*2Pv** |
| #268 | Del | arr[hg19] 2q13(110,504,318-111,365,996)x1 | 861,678 | 16 | 3 | NPH1 | ASD | M/- |
| #276 | Dup | arr[hg19] Xq26.2(130,672,818-130,967,726)x3 | 294,908 | 2 | 3 | KAL1 | DD, FD, cardiomyopathy, thyroid dysfunction and myopia | **F/-** |
| #278 | Dup | arr[hg19] 19q13.42(54,201,711-54,420,807)x3 | 219,096 | 39 | 9 | MIR, NLRP12 | Epilepsy, abnormal brain structure | **F/-** |
| #290 | Dup | arr[hg19] 2q13(110,496,601-110,983,418)x3 | 486,817 | 14 | 3 | NPHP1 | Genetic counseling | **M/-** |
| #294 | Dup | arr[hg19] 2q13(110,498,141-110,980,295)x3 | 482,154 | 14 | 3 | NPHP1 | DD, ID, FD and | **F/-** |
| #299 | Dup | arr[hg19] 17q11.2(28,952,286-29,150,025)x3 | 197,739 | 4 | 1 | CRLF3 | DD, ASD and behavioural disorder | M/- |
| #309 | Del | arr[hg19] 17p13.1(6,949,507-7,217,381)x1 | 267,874 | 16 | 15 | - | Short stature, DD, ID, FD and microcephaly | **M/-** |
| #311 | Dup | arr[hg19] 1p31.3(61,699,736-62,125,970)x3 | 426,234 | 2 | 1 | NFIA | Obesity, CAs, DD, | **F/-** |
| #319 | Dup | arr[hg19] 16p13.3(1,252,411-1,404,818)x3 | 152,407 | 9 | 8 | 5 OMIMs | Anal imperforation, onfalocele and cloacal exstrophy | **F/-** |
| #331 | Dup | arr[hg19] 4p16.3p16.2(4,025,257-4,618,896)x3 | 593,639 | 7 | 3 | NSG1 | DD, epilepsy and FD | **M/*Pv** |
| #336 | Dup | arr[hg19] 1q25.3(183,589,206-183,827,325)x3 | 238,119 | 3 | 3 | ARPC5, APOBEC4, RGL1 | DD and FD | **F/-** |
| #342 | Del | arr[hg19] 3p24.2(24,376,230-24,492,572)x1 | 116,342 | 1 | 1 | THRB | DD, Bilateral hearing impairment and FD | **F/-** |
| #346 | Del | arr[hg19] 7q31.1(111,485,313-111,922,531)x1 | 437,218 | 2 | 2 | DOCK4 | Low weight, slender build, motor delay, DD, SLD, SevID and ASD. | M/- |
| #354 | Dup | arr[hg19] 9q33.1(118,409,943-119,207,073)x3 | 797,13 | 4 | 3 | NOC2L | Consanguineous parents, quadriparesis, DD, FD and ostium secundum | **M/-** |
| #359 | Dup | arr[hg19] 5q14.1(80,019,759-80,535,750)x3 | 515,991 | 6 | 3 | MSH3, RASGRF2, CKMT2 | Convulsions, LDO, MID | **F/-** |
| #360 | Del | arr[hg19] 1p31.1(72,257,666-72,499,784)x1 | 242,118 | 2 | 1 | NEGR1 | Convulsions, LDO, F | **F/-** |
| #369 | Dup | arr[hg19] 12p11.22p11.21(30,175,955-31,570,927)x3 | 1,394 | 9 | 3 | IPO8, CAPRIN2, DDX11 | Abnormal brain structure | **M/-** |
| #383 | Dup | arr[hg19] 10q11.23(51,250,417-51,755,110)x3 | 504,693 | 7 | 4 | PARG , MSMBP , NCOA4 , TIMM23 | Convulsions, DD, SLD, ASD, behavioural disorder and gluten intolerance | M/- |
| #384 | Dup | arr[hg19] 10q21.1(59,984,568-60,285,875)x3 | 301,307 | 5 | 5 | IPMK, CISD1, UBE2D1 | Motor delay and chronic encephalopathy | **M/-** |
| #384 | Dup | arr[hg19] 18q22.3(72,755,482-73,023,597)x3 | 268,115 | 3 | 1 | TSHZ1 | Motor delay and chronic encephalopathy | **M/-** |
| #397 | Dup | arr[hg19] 16p12.2(21,817,921-22,431,357)x3 | 613,436 | 9 | 3 | UQCRC2, EEF2K, CDR2 | DD, ASD and FD | M/- |
| #401 | Dup | arr[hg19] 2q11.1(95,733,867-96,279,208)x3 | 545,341 | 8 | 3 | ZNF2, MRPS5, KCNIP3 | ASD, DF | F/- |
| #423 | Dup | arr[hg19] 12q21.31(80,559,698-80,918,615)x3 | 358,917 | 2 | 2 | OTOGL, PTPRQ | CAs, ID and FD | **F/-** |
| #444 | Del | arr[hg19] 16p13.3(6,644,079-6,675,606)x1 | 31 | 1 | 1 | RBFOX1 | ASD | **M/-** |
| #456 | Dup | arr[hg19] 8q24.3(144,262,042-144,486,369)x3 | 224 | 8 | 4 | GPIHBP1 | ASD and SLD | M/- |
| #477 | Dup | arr[hg19] 7p15.3(24,133,960-24,671,640)x3 | 538 | 2 | 2 | NPY, MPP6 | ASD and ID | M/- |
| #492 | Dup | arr[hg19] Yq11.221q11.222(19,563,599-21,028,944)x4 | 1,465 | 12 | 3 | XKRY, HSFY1, CDY2A | ASD and ID | M/ 46, XY, inv(9) |
| #500 | Del | arr[hg19]10q23.2(88,466,260-88,577,094)x1 | 111 | 2 | 2 | LDB3 (605906), BMPR1A (601299) | Multiple CAs, right periorbital oedema, thumbs with distal implantation, nail hypoplasia, absence of distal phalanx,  bilateral 5^th^ finger hypoplasia, single unilateral palmar fold, clubfoot to the right, omphalocele,  pulmonary cardiopathy | F/- |
| #567 | Dup | arr[hg19]16p13.3(1,052,880-1,268,271)x3 | 215 | 4 | 3 | C1QTNF8 (*614147), CACNA1H (*607904) | Short stature, disturbed  behaviour, long palpebral fissures, long eyelashes, long philtrum, anteverted nostrils, posteriorly rotated ears, short fingers, finger pads, partial syndactyly, wide hallux, dysplastic nails of 2^nd^ and 5^th^ toes, hyperlordosis. | M/- |
| #577 | Dup | arr[hg19]4q28.1(126,172,904-126,474,296)x3 | 301 | 2 | 1 | FAT4 (612411) | DD, CNS malformation, cerebellar hypoplasia, lack of CP growth | M/- |
| #584 | Dup | arr[hg19]4q21.1q21.21(78,742,412-78,989,716)x3 | 247 | 2 | 2 | MRPL1 (611821), FRAS1 (607830) | DD, macrocephaly, FD | M/- |
| #592 | Dup | arr[hg19] 9q33.1(119,677,859-120,377,754)x3 | 700 | 2 | 1 | ASTN2 | ASD, ID, SLD and hemiparesis | F/- |
| #595 | Dup | arr[hg19] 14q12(32,110,535-32,560,537)x3 | 450 | 4 | 2 | NUBPL(*613621) | ASD, seizures | M/- |
| #602 | Del | arr[hg19]Xq25(126,962,874-127,374,779)x1 | 412 | 1 | 1 | ACTRT1 (*300487) | Suspected Alagille syndrome | P/- |
| #607 | Dup | arr[hg19] 8q24.3(143,610,752-143,933,329)x3 | 322 | 15 | 9 | SLURP1 (*606119) | Macrocephaly, advanced bone age, skin spots, ectrodactilia | F/- |
| #635 | Dup | arr[hg19]16p12.2(21,841,354-22,442,007)x3 | 600 | 13 | 4 | UQCRC2 (*191329) | Atrophy, polymicrogyria, lissencephaly, microcephaly, interatrial communication, DD | F/ 2VUS |
| #635 | Dup | arr[hg19]2q21.1(131,502,025-131,970,782)x3 | 468 | 5 | 3 | ARHGEF4 (*605216) | Atrophy, polymicrogyria, lissencephaly, microcephaly, interatrial communication, DD | F/ 2VUS |
| #644 | Dup | arr[hg19]15q15.3(43,868,571-43,977,181)x3 | 108 | 5 | 4 | STRC (606440) | DD, suspected Susac syndrome | M/- |
| #646 | Dup | arr[hg19]13q33.2q34(105,943,388-114,027,457)x3 | 8,084 | 68 | 21 | - | Leopard syndrome like frekles, mildID,  alopecia, tremor | F/ +1PCNV |
| #649 | Dup | arr[hg19] 7q21.3(96,630,285-96,773,686)x3 | 143 | 3 | 3 | DLX5 (600028) | Multiple CAs, cardiopathy, bilateral thumb agenesis, vertical talus feet, microtia, suspected TAR or VACTERL | F/ 3VUS |
| #649 | Dup | arr[hg19] Xq21.1(76,959,491-77,184,107)x3 | 224 | 4 | 4 | COX7B (300885) | Multiple CAs, cardiopathy, bilateral thumb agenesis, vertical talus feet, microtia, suspected TAR or VACTERL | F/ 3VUS |
| #649 | Dup | arr[hg19] Xp11.3 (44,597,231-44,820,429)x3 | 223 | 2 | 2 | KDM6A (*300128) | Multiple CAs, cardiopathy, bilateral thumb agenesis, vertical talus feet, microtia, suspected TAR or VACTERL | F/ 3VUS |
| #658 | Dup | arr[hg19] 9q22.31(94,665,153-94,812,523)x3 | 147 | 2 | 2 | ROR2, SPTLC1 | ASD | M/2VUS |
| #658 | Dup | arr[hg19] 13q12.3(31,764,937-32,190,263)x3 | 425 | 1 | 1 | B3GLCT | ASD | M/2VUS |
| #661 | Dup | arr[hg19] 6q14.3(87,410,108-87,911,748)x3 | 502 | 3 | 3 | HTR1E, CGA, ZNF292 | ASD | M/Brother with ASD #659 (negative) |
| #671 | Dup | arr[hg19] 14q12(29,221,762-29,459,916) x3 | 238 | 4 | 1 | FOXG1 (*164874) | Epilepsy, DD, suspected Dravet syndrome (SCN1A) | F/- |
| #695 | Del | arr[hg19] 16p13.3(6,887,840-6,966,572)x1 | 79 | 1 | 1 | RBFOX1 | ASD | M/- |
| #716 | Dup | arr[hg19] 3p21.31 (45779135-46005169)x3 | 226 | 6 | 6 | SLC6A20  (*605616) | Disturbed behavior, SLD, FD, Karyotype: 46,XY,22ps + | M/- |
| #727 | Dup | arr[hg19] 2q13(110,504,318-111,365,996)x3 | 862 | 16 | 3 | NPHP1 | ASD, DD, abnormal growth, FD, CAs, macrocephaly and hirsutism | F/- |
| #738 | Dup | arr[hg19] 15q13.3(31,999,631-32,444,043)x3 | 444 | 1 | 1 | CHRNA7 | ASD, ID, FD, SLD, abnormal growth, dyslalia, motor difficulties and ADHD | M/- |
| #741 | Dup | arr[hg19]13q12.12(23,473,290-24,979,038)x3 | 1,506 | 15 | 8 | - | Multiple comorbidities  (not specified) | M/- |
| #742 | Dup | arr[hg19] 14q12(32,110,535-32,600,382)x3 | 490 | 4 | 2 | NUBPL (*613621) | Difficult-to-control epilepsy,  cerebral palsy, DD, overweight,  frontal temporal cortical dysplasia | M/- |
| #744 | Dup | arr[hg19] 17p13.3(791,201-977,024)x3 | 186 | 5 | 3 | TIMM22 (*607251) e ABR (*600365) | ASD, hypertension,  dyslipidemia | M/- |
| #756 | Del | arr[hg19]6p22.3(17,808,245-17,978,255)x1 | 170,011 | 1 | 1 | KIF13A (605433) | Deformed toes, clubbed fingers, ptosis, proeminent nose, kyphosis, short stature, DD | M/- |
| #758 | Dup | arr[hg19]15q13.3(32,003,538-32,931,921)x3 | 928 | 12 | 8 | CHRNA7 (*118511) | DD, hypotonia, strabismus, | F/- |
| #761 | Del | arr[hg19] Xp22.31(6,455,149-8,135,644)x1 | 1,680 | 5 | 5 | STS (*300747) | Failure to thrive, DD | F/- |
| #768 | Dup | arr[hg19] 1p36.32(3,311,950-3,589,407)x3 | 277 | 8 | 6 | PRDM16 (*605557) | Arched palate, hair line in M, wide-spaced nipples, heart fremitus, cubitus valgus,  growth delay | F/- |
| #769 | Dup | arr[hg19]18q21.1(44,515,228-44,898,600)x3 | 383 | 6 | 4 | KATNAL2  (*614697) | dystonia, dysarthria, hyperactivity, mild ID | F/- |
| #777 | Del | arr[hg19]7q34(141,799,147-142,047,384)x1 | 248 | 5 | 1 | MGAM (154360) | congenital deafness | M/ brother of  #778 |
| #777 | Dup | arr[hg19]7p15.2(25,994,970-26,238,444)x3 | 244 | 2 | 2 | NFE2L3 (604135), HNRNPA2B1 (600124) | congenital deafness | M/ brother of  #778 |
| #778 | Del | arr[hg19]7q34(141,799,147-142,047,384)x1 | 248 | 5 | 1 | MGAM (154360) | congenital deafness | M/ brother of  #777 |
| #778 | Dup | arr[hg19]7p15.2(25,994,970-26,238,444)x3 | 244 | 2 | 2 | NFE2L3 (604135), HNRNPA2B1 (600124) | congenital deafness | M/ brother of  #777 |
| #785 | Dup | arr[hg19]9p24.3(208,455-336,687)x3 | 128 | 2 | 1 | DOCK8 (611432) | single palmar fold, inverted nipples, bilateral cryptorchidism, inguinal hernia, growth delay | M/- |
| #790 | Del | arr[hg19] 1p13.3(108,700,187-108,962,439)x1 | 262 | 4 | 2 | SLC25A24 (608744) | cleft palate, DD, FD, hyperactivity | M/- |
| #792 | Del | arr[hg19] 1p32.1(59,848,744-60,044,036)x1 | 195 | 1 | 1 | FGGY (*611370) | Mild DD, generalised epilepsy, mild tricuspid insufficiency, right lung hypoplasia | F/- |
| #798 | Del | arr[hg19] 4q13.3q21.1(74,958,696-76,339,793)x1 | 1,381 | 13 | 7 | - | consanguineous parents,  pre and post-natal short stature, microcephaly,  hypertrophic cardiomyopathy,  oblique palpebral fissures | F/- |
| #810 | Dup | arr[hg19] 7p11.2(57,233,082-57,906,704)x3 | 674 | 4 | - | - | ASD | M/- |
| #814 | Dup | arr[hg19] 15q11.2(22,770,421-23,288,350)x3 | 518 | 8 | 4 | NIPA1 | ASD | M/- |
| #816 | Del | arr[hg19] 8q21.13(80,370,864-82,083,687)x1 | 1,713 | 10 | 5 | - | DD, cardiac malformation, global hypotonia, signs of speech apraxia | M/- |
| #828 | Del | arr[hg19]5p15.31p15.2(9,090,338-11,635,988)x1 | 2,545 | 20 | 8 | - | ID, strabismus, protruding ears  brother of case #829 | M/ +1 PCNV |
| #829 | Del | arr[hg19]5p15.31p15.2(9,090,338-11,635,988)x1 | 2545 | 20 | 8 | - | ID and DF | M/ Brother of case #828 + 1 PCNV |
| #830 | Del | arr[hg19] 11p14.2p14.1(26,997,314-27,233,664)x1 | 236 | 3 | 1 | BBOX1 | ASD | M/- |
| #830 | Del | arr[hg19] 11p14.2p14.1(26,997,314-27,233,664)x1 | 236 | 3 | 1 | BBOX1 | ASD | M/- |
| #835 | Del | arr[hg19]16p13.3(6,887,841-7,013,897)x1 | 126 | 1 | 1 | RBFOX1(*605104) | ModID, DD, divergent strabismus, scoliosis | F/- |
| #835 | Dup | arr[hg19]11p15.2(14,589,177-15,240,408)x3 | 651 | 6 | 6 | - | ModID, DD, divergent strabismus, scoliosis | F/- |
| #854 | Dup | arr[hg19] 1q21.1(145,369,184-145,988,238)x3 | 619 | 24 | 12 | RBM8A (605313), PEX11B (603867) | Developmental regression, seizures,  suspected Rett syndrome. | F/- |
| #855 | Dup | arr[hg19] 14q12(26,490,666-27,520,832)x3 | 1,057 | 6 | 1 | NOVA1 (*602157) | ASD | F/- |
| #870 | Dup | arr[hg19] 12p11.1(34,065,100-34,802,951)x3 | 738 | 2 | 1 | ALG10 (*603313) | Ataxic hypotonic cerebral palsy, cannot walk, absent speech, seizures | F/- |
| #876 | Dup | arr[hg19] Xq13.1(69,207,741-69,317,932)x3 | 110 | 3 | 3 | EDA (300451) | Macrocephaly, broad forehead,  sialorrhea, limb hemihypertrophy, joint laxity, suspected Sotos syndrome | F/- |
| #880 | Del | arr[hg19] 16p13.3(6,967,626-6,998,141)x1 | 30,5 | 1 | 1 | RBFOX1 (* 605104) | ASD | M/- |
| #904 | Del | arr[hg19] 7q31.1(110,863,879-111,283,978)x1 | 420 | 1 | 1 | IMMP2L | ASD and DD | M/- |
| #914 | Dup | arr[hg19]3p14.3(57,370,900-57,746,279)x3 | 375 | 7 | 4 | - | ID, microcephaly, cleft lip, foot anomaly,  small hands, ichthyosis vulgaris | F/- |
| #918 | Dup | arr[hg19]10p15.1(4,620,457-5,104,391)x3 | 484 | 7 | 4 | - | ADHD, ID | M/- |
| #927 | Dup | arr[hg19]15q11.2(22,770,422-23,615,769)x3 | 845 | 4 | 4 | - | Suspected Angelman syndrome | M/ 1 PCNV |
| #927 | Dup | arr[hg19] 15q11.2(22,770,421-23,615,769)x3 | 845 | 16 | 4 | - | Suspected Angelman syndrome | M/ 1 PCNV |
| #928 | Del | arr[hg19] 1q21.2(147,723,034-147,830,830)x1 | 108 | 2 | 1 | NBPF8 | ASD, FD, SLD, CAs and macrocephaly | M/- |
| #929 | Dup | arr[hg19] 8p21.3(19,775,847-20,071,770)x3 | 296 | 3 | 3 | LPL (*609708) | Fetal losses with malformation | F/ 2 VUS |
| #929 | Dup | arr[hg19] 11q14.1(77,492,774-78,509,705)x3 | 1,017 | 16 | 3 | - | Fetal losses with malformation | F/ 2 VUS |
| #937 | Del | arr[hg19] 1q41(219,090,413-219,734,998)x1 | 645 | 4 | 1 | LYPLAL1 | ASD | M/3VUS |
| #937 | Dup | arr[hg19] 1q41q42.12(221,795,858-225,581,420)x3 | 3,786 | 29 | 13 | WDR26 | ASD | M/3VUS |
| #937 | Dup | arr[hg19] Xq21.1(76,993,262-77,392,096)x2 | 399 | 7 | 7 | - | ASD | M/3VUS |
| #941 | Dup | arr[hg19] Xq28(152,927,530-152,991,389)x2 | 64 | 4 | 4 | ABCD1 | ASD, MildID, SLD and hyperkinetic disorder | M/- |
| #942 | Dup | arr[hg19]Xp21.2(31,116,865-31,359,757)x3 | 243 | 1 | 1 | DMD (*300377). | DD, SLD, hiperactivity,  oppositional defiant disorder,seizures | F/- |
| #954 | Del | arr[hg19] 16p13.3(3,315,091-3,432,025)x1 | 117 | 6 | 3 | - | ASD, DD, Abnormal growth, FD, CAs, abnormal brain structure, motor difficulties and epilepsy | M/- |
| #959 | Dup | arr[hg19] 2q12.2q12.3(106,873,992-108,480,894)x3 | 1,607 | 9 | 4 | - | Suspected genetic condition | F/- |
| #984 | Del | arr[hg19] 13q22.2q31.1(76,555,343-80,068,154)x1 | 3,513 | 24 | 12 | - | ASD, motor agitation and use of corrective lenses | M/- |
| #1006 | Del | arr[hg19] 15q11.2(22,770,421-23,214,655)x1 | 444 | 6 | 4 | CYFIP1, NIPA2 | ASD | M/- |
| #1021 | Dup | arr[hg19]3p24.1(30577491-30775657)x3 | 198 | 2 | 2 | TGFBR2 (*190182) | microcephaly and micrognathia | M/- |
| #1053 | Del | arr[hg19] 7q31.1(111,483,652-111,922,578)x1 | 439 | 4 | 2 | DOCK4 e ZNF277 | ASD, midID, obesity and gynecomastia | M/- |
| #1057 | Dup | arr[hg19] 8q12.1(56,717,039-57,240,751)x3 | 523 | 10 | 7 | CHCHD7 (*611238) | short stature,  limb anomalies | F/- |
| #1088 | Dup | arr[hg19] 1q43(237,843,614-238,415,416)x3 | 572 | 3 | 2 | RYR2 (180902), ZP4 (613514) | DD, ataxia,  psychomotor agitation | M/- |
| #1091 | Dup | arr[hg19] Xq11.2q12(64,008,668-64,866,293)x2 | 858 | 4 | 3 | ZC4H2 | ASD | M/- |
| #1099 | Dup | arr[hg19] 16q24.1(84,439,871-84,676,492)x3 | 237 | 4 | 2 | ATP2C2 (*613082) | Obesity, ASD,  Karyotype: 46,XY inv3(q13q26) | F/- |
| #1108 | Dup | arr[hg19] 15q11.2(22,770,421-23,082,237)x3 | 312 | 6 | 4 | NIPA1 (*608145) | poor motor coordination, DD, SLD, anxiety | F/- |
| #1109 | Dup | arr[hg19]4p16.1(7,136,707-11,214,146)x3 | 4,077 | 47 | 17 | - | Additional material on chromosome 3. | M/ +1 PCNV |
| #1119 | Dup | arr[hg19] 2p13.2(71,586,200-71,730,470)x3 | 144 | 2 | 2 | ZNF638 (614349), DYSF (603009). | ID, syndromic, epilepsy, scoliosis | F/- |
| #1120 | Dup | arr[hg19] 15q21.1(45,225,243-45,806,154)x3 | 581 | 26 | 12 | - | ASD and MildID | M/- |
| #1127 | Del | arr[hg19]2q31.2(179,396,924-179,629,278)x1 | 232 | 2 | 2 | TTN | ASD and epilepsy | M/ |

Copy Number Variants of Unknown Significance (VUS) found in the cohort, with the number of genes present in the region, listing the most relevant genes and phenotypes for each individual. Dup = Duplication, Del = Deletion, CAs = Congenital Anomalies, DD = Developmental Delay, ID = Unspecified intellectual disability, MildID  = Mild Intellectual Disability, ModID = Moderate Intellectual Disability, SevID = Severe Intellectual Disability, Aut = Autism, and FD = Facial Dysmorphisms, SLD = speech and/or language delay or impairment, IUGR = Intrauterine growth restriction, ADHD = Attention-deficit/hyperactivity disorder, LDO = learning difficulty only (no ID), ASD = Autism spectrum disorder, F = Female, M = Male. LPCNVs = likely pathogenic CNVs. *2V = Patients with 2 VUS. *3V = Patients with 3 VUS.

1. Supplementary table 2

**Prevalence of Syndromes Associated with Pathogenic CNVs.**

| Identified syndromes | Frequency in the cohort (%) | Frequencies in pathogenic CNVs (%) |
| --- | --- | --- |
| Angelman/Prader Willi syndrome | 0,69 | 3,38 |
| Di George syndrome | 0,69 | 3,38 |
| 1p36 deletion syndrome | 0,59 | 2,90 |
| 16p11.2 deletion syndrome | 0,49 | 2,42 |
| Cri Du Chat syndrome | 0,49 | 2,42 |
| Inverted 8p duplication/deletion syndrome | 0,49 | 2,42 |
| 1q21.1 Microduplication Syndrome | 0,39 | 1,93 |
| 22q11.2 duplication syndrome | 0,39 | 1,93 |
| Distal 13q deletion Syndrome | 0,39 | 1,93 |
| partial trisomy 7q31.32q33 | 0,39 | 1,93 |
| PhelanMcDermid Syndrome | 0,39 | 1,93 |
| Waardenburg syndrome | 0,39 | 1,93 |
| Williams Beuren Syndrome | 0,39 | 1,93 |
| 2q37 Microdeletion Syndrome | 0,29 | 1,45 |
| Koolen de Vries syndrome | 0,29 | 1,45 |
| partial trisomy 19p13 | 0,29 | 1,45 |
| Terminal 21q del | 0,29 | 1,45 |
| Trissomia Parcial do 21q | 0,29 | 1,45 |
| 15q11q13 duplication syndrome | 0,20 | 0,97 |
| 15q13.3 Microdeletion Syndrome | 0,20 | 0,97 |
| 18p deletion syndrome | 0,20 | 0,97 |
| 18q deletion syndrome | 0,20 | 0,97 |
| 2q31.1 microdeletion syndrome | 0,20 | 0,97 |
| 6p25 deletion syndrome | 0,20 | 0,97 |
| 9p Deletion Syndrome | 0,20 | 0,97 |
| ATR16 syndrome | 0,20 | 0,97 |
| Distal 16p11.2 deletion Syndrome | 0,20 | 0,97 |
| partial 13q monosomy syndrome | 0,20 | 0,97 |
| Partial Trisomy Distal 4q | 0,20 | 0,97 |
| Temple syndrome | 0,20 | 0,97 |
| Trisomy 12p | 0,20 | 0,97 |
| 8p23.1 deletion syndrome | 0,10 | 0,48 |
| 10q26 deletion syndrome | 0,10 | 0,48 |
| 15q24 deletion syndrome | 0,10 | 0,48 |
| 16p13.3 microduplication syndrome | 0,10 | 0,48 |
| 18 q21.32qter deletion syndrome | 0,10 | 0,48 |
| 21q22.12 microdeletion syndrome | 0,10 | 0,48 |
| 3q29 Microduplication syndrome | 0,10 | 0,48 |
| 7q3436 deletion syndrome | 0,10 | 0,48 |
| 8p intersticial deletion including p12 syndrome | 0,10 | 0,48 |
| CharcotMarieTooth disease type 1A – CMT1A ( | 0,10 | 0,48 |
| CoffinSiris syndrome | 0,10 | 0,48 |
| Cohen syndrome | 0,10 | 0,48 |
| distal trisomy 10q syndrome | 0,10 | 0,48 |
| Distal trisomy 18q | 0,10 | 0,48 |
| Distal trisomy 3q | 0,10 | 0,48 |
| Distal trisomy 8p | 0,10 | 0,48 |
| DYRK1A related intellectual disability syndrome | 0,10 | 0,48 |
| Greig syndrome | 0,10 | 0,48 |
| KBG syndrome | 0,10 | 0,48 |
| Keutel syndrome | 0,10 | 0,48 |
| MECP2 Duplication Syndrome | 0,10 | 0,48 |
| partial 18p deletion syndrome | 0,10 | 0,48 |
| Partial Trisomy 16p13.3 Syndrome | 0,10 | 0,48 |
| partial trisomy 18q | 0,10 | 0,48 |
| partial trisomy 5p14.3p15.31 | 0,10 | 0,48 |
| Rett syndrome | 0,10 | 0,48 |
| Schinzel Giedion Syndrome | 0,10 | 0,48 |
| SimpsonGolabiBehmel Syndrome | 0,10 | 0,48 |
| Síndrome de San Luis Valley | 0,10 | 0,48 |
| Síndrome Tricorrinofalangeana (TRPS). | 0,10 | 0,48 |
| SmithMagenis Syndrome | 0,10 | 0,48 |
| Sotos Syndrome | 0,10 | 0,48 |
| tetrasomy 18p11.21p11.32 | 0,10 | 0,48 |
| Triple X Syndrome | 0,10 | 0,48 |
| WAGR syndrome | 0,10 | 0,48 |
| WolfHirschhorn syndrome | 0,10 | 0,48 |
| Xq26.3, Xq27.3q28 and Xq28 duplication syndromes | 0,10 | 0,48 |
| Xq27.3q28 and Xq28 duplication syndrome | 0,10 | 0,48 |
| Xq28 duplication syndrome | 0,10 | 0,48 |
| XYYRegion Syndrome | 0,10 | 0,48 |
| Total syndromes identified  Note: Values represent the prevalence of syndromes in individuals with idenified pathogenic CNVs. | **14,51** | **71,50** |

1.
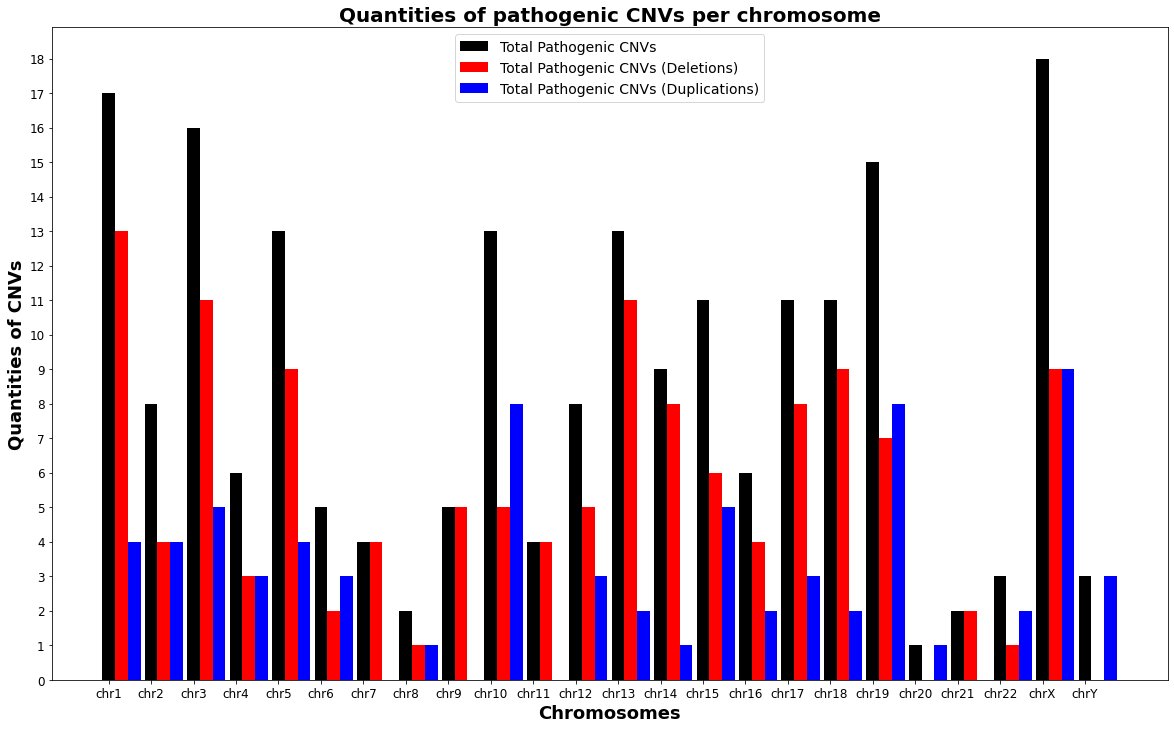
Supplementary information 1


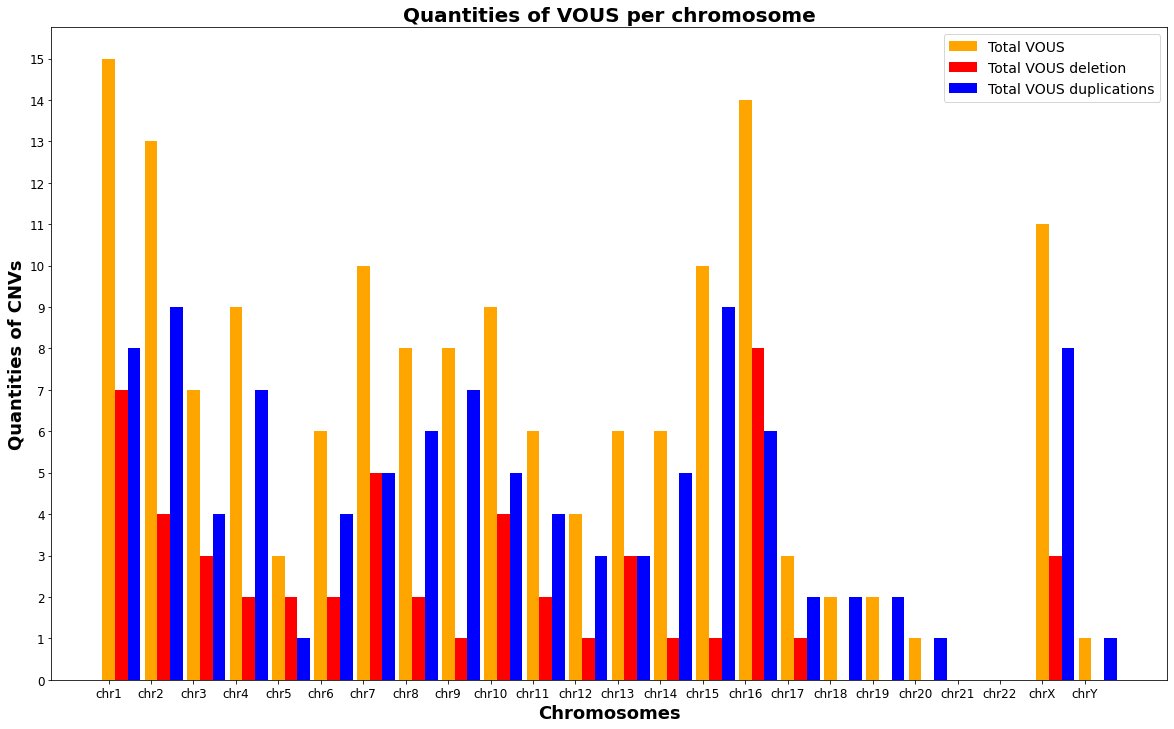
Pathogenic CNVs per chromosome

VUS per chromosome.

1. Supplementary information 2

**Comparison of CNVs interpreted, by size X number of genes covered (A & B) and by size X number of OMINs genes covered (C & D):**


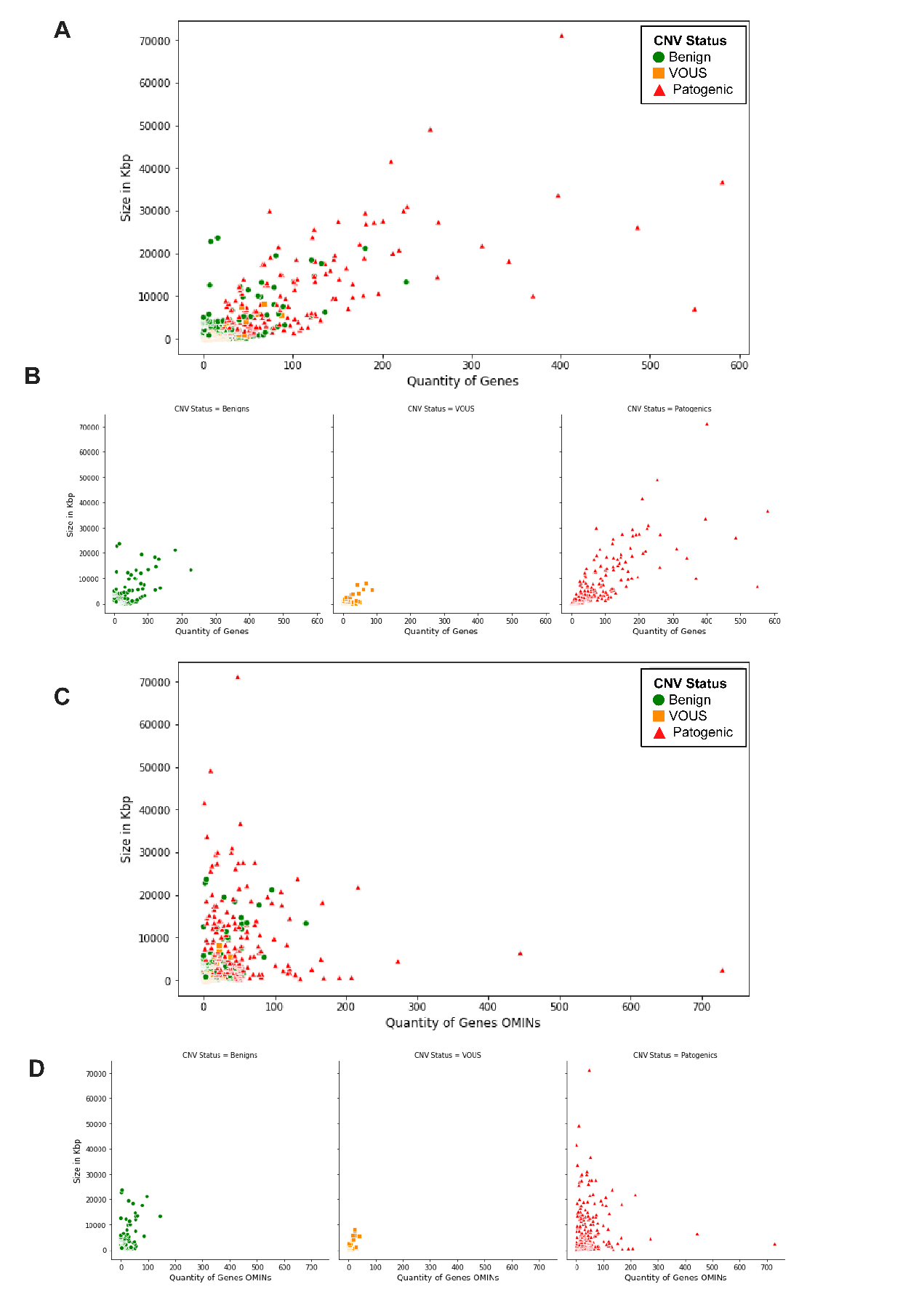


1. Supplementary information 3


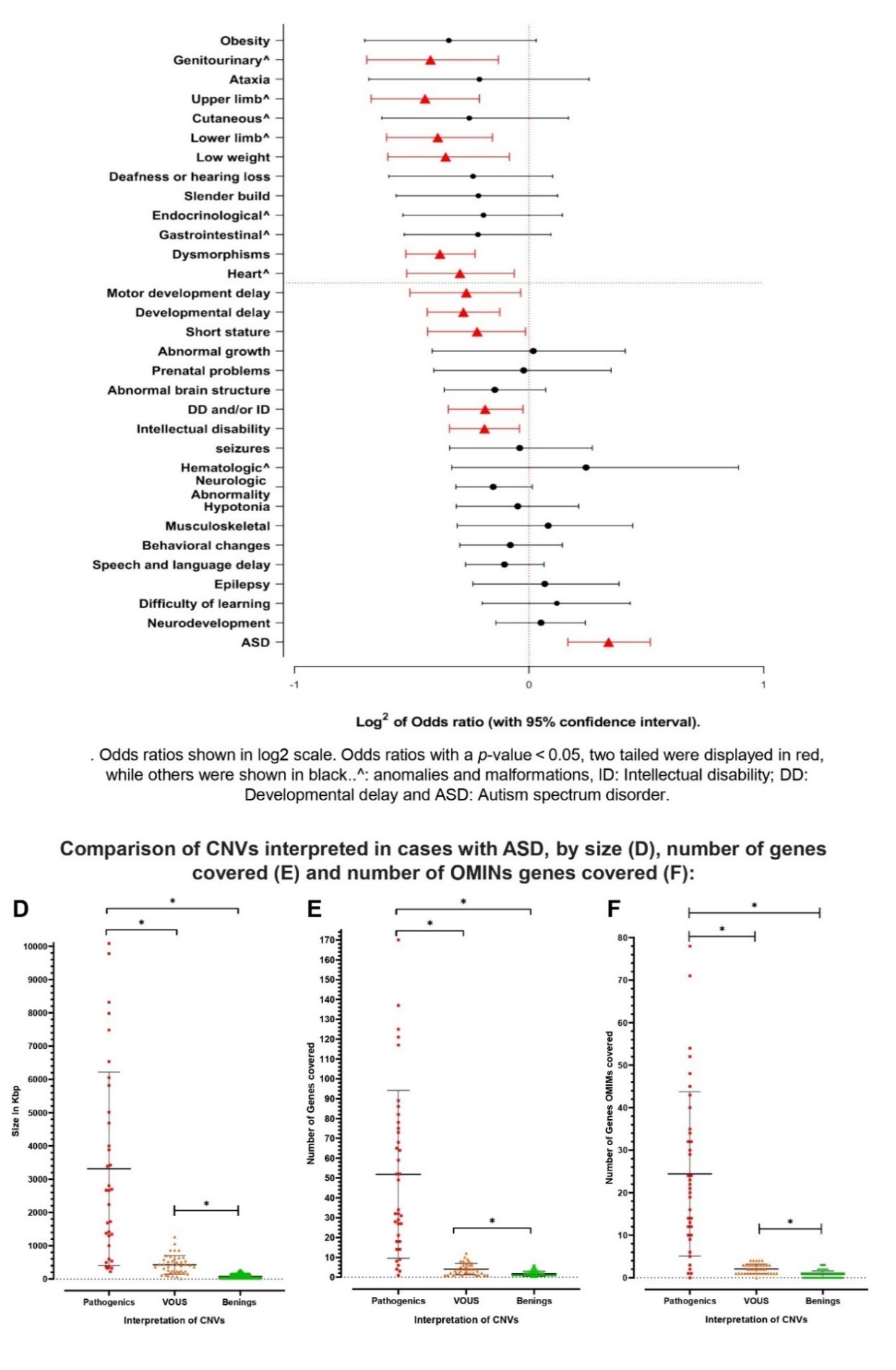


1. Supplementary information 4

**Circus ideogram with pathogenic CNVs found in ASD cases, plotted by chromosome**.


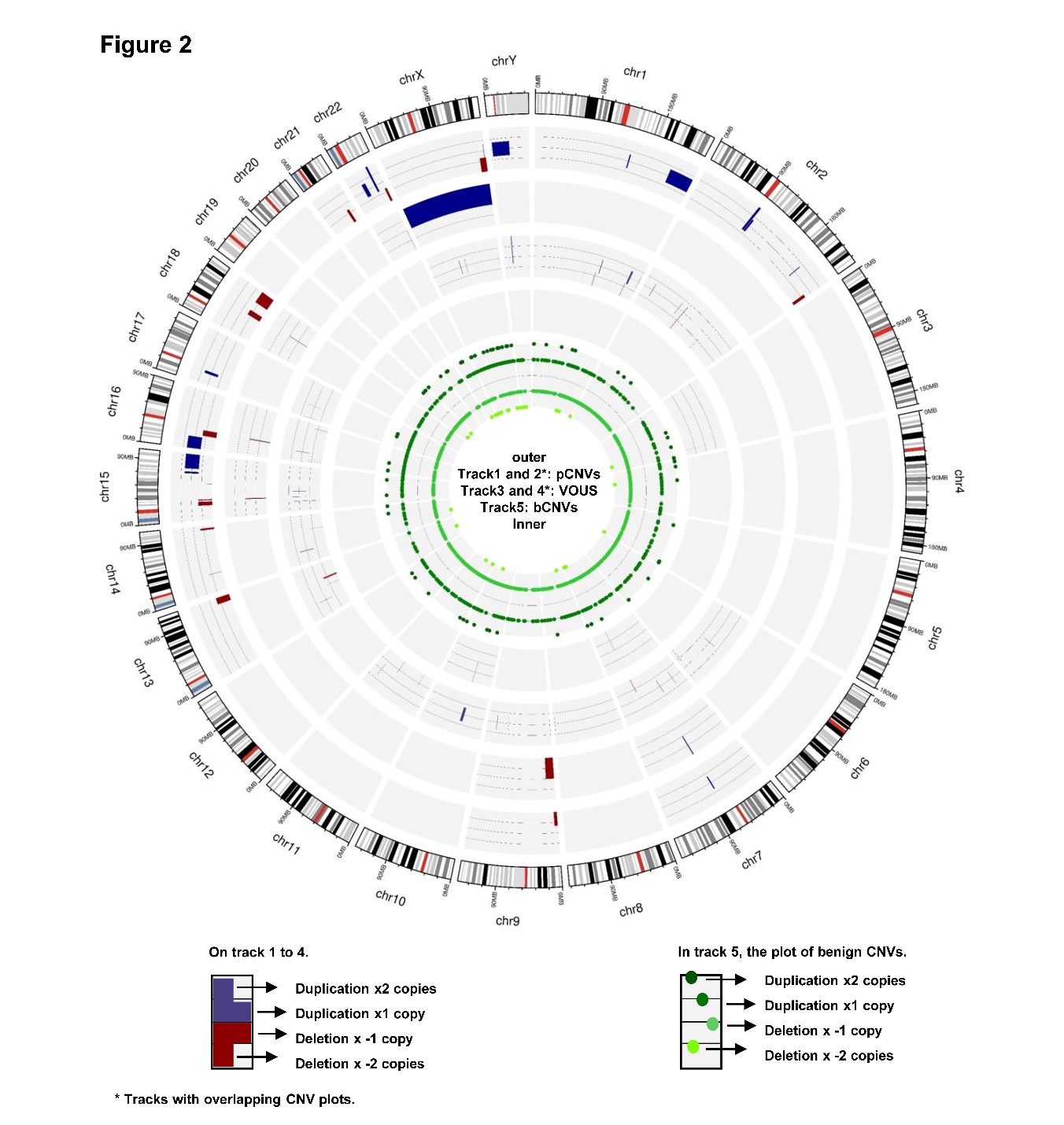


Figure 2: The circle ideogram plot shows in the ouhermost tracks the genomic positions of all pathogenic CNVs (pCNVs) found in the study per human chromosome, wich are plotted in two tracks, the first and the second to allow visualization of overlapping pCNVs. The VUS are plotted in the third and fourth tracks (also two tracks, for overlapping VUS). The bars in blue represent duplication, either for x1 or x2 additional copies. The bars in red represent deletions in x1 (heterozygous) or x2 (hemozygous) copies. At the center of track 5 are plotted all the CNVs interpreted as benign (bCNVs) detected in the study, these in turn, are presented by dots in shades of green, which represent different states of copies (duplication x1 or x2 copies and deletions x1 or x2 copies).
